# Advancements and prospects of perovskite-based fuel electrodes in solid oxide cells for CO_2_ electrolysis to CO

**DOI:** 10.1039/d4sc03306j

**Published:** 2024-06-27

**Authors:** Ruijia Xu, Shuai Liu, Meiting Yang, Guangming Yang, Zhixin Luo, Ran Ran, Wei Zhou, Zongping Shao

**Affiliations:** a State Key Laboratory of Materials-Oriented Chemical Engineering, College of Chemical Engineering, Nanjing Tech University Nanjing 211816 China ygm89525@njtech.edu.cn zongping.shao@curtin.edu.au; b WA School of Mines: Minerals, Energy & Chemical Engineering (WASM-MECE), Curtin University Perth WA 6102 Australia

## Abstract

Carbon dioxide (CO_2_) electrolysis to carbon monoxide (CO) is a very promising strategy for economically converting CO_2_, with high-temperature solid oxide electrolysis cells (SOECs) being regarded as the most suitable technology due to their high electrode reaction kinetics and nearly 100% faradaic efficiency, while their practical application is highly dependent on the performance of their fuel electrode (cathode), which significantly determines the cell activity, selectivity, and durability. In this review, we provide a timely overview of the recent progress in the understanding and development of fuel electrodes, predominantly based on perovskite oxides, for CO_2_ electrochemical reduction to CO (CO_2_RR) in SOECs. Initially, the current understanding of the reaction mechanisms over the perovskite electrocatalyst for CO synthesis from CO_2_ electrolysis in SOECs is provided. Subsequently, the recent experimental advances in fuel electrodes are summarized, with importance placed on perovskite oxides and their modification, including bulk doping with multiple elements to introduce high entropy effects, various methods for realizing surface nanoparticles or even single atom catalyst modification, and nanocompositing. Additionally, the recent progress in numerical modeling-assisted fast screening of perovskite electrocatalysts for high-temperature CO_2_RR is summarized, and the advanced characterization techniques for an in-depth understanding of the related fundamentals for the CO_2_RR over perovskite oxides are also reviewed. The recent pro-industrial application trials of the CO_2_RR in SOECs are also briefly discussed. Finally, the future prospects and challenges of SOEC cathodes for the CO_2_RR are suggested.

## Abstract 2

Developments and prospects for solid oxide cells using a perovskite-based fuel electrode for CO_2_ electrolysis to CO.

## Graphical abstract

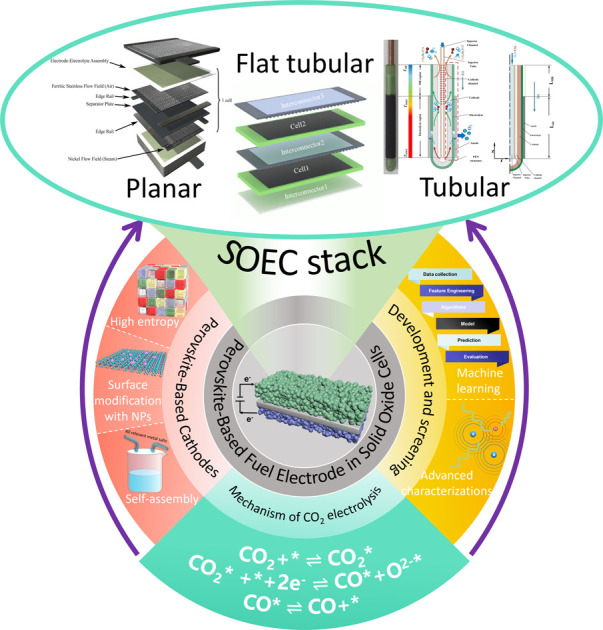

## 1. Introduction

Our current world is over-dependent on fossil fuels to satisfy global energy demands, resulting in substantial CO_2_ emissions. With the increasing concerns about healthy energy supply and sustainable ecological and environmental systems, the world is looking for a more sustainable energy system. Many countries have set targets to achieve net-zero carbon dioxide emissions by the mid-21st century, emphasizing the need to establish a clean, low-carbon, secure, and efficient energy infrastructure.^1^

There are mainly three ways to realize a net-zero CO_2_ emissions target, *i.e.*, the use of renewable energy/hydrogen energy, CO_2_ sequestration, and CO_2_ re-utilization. By developing the latter two technologies, fossil fuels can still be used to achieve the net-zero CO_2_ emissions target, while renewable energy can serve as an energy input for CO_2_ sequestration and re-utilization. This approach is highly attractive since the current infrastructure for fossil fuel production, storage and utilization is highly matured and can be continuously used, while the net-zero CO_2_ emissions target is reached due to the storage or recycling of the CO_2_ produced. Consequently, the development of efficient CO_2_ storage and conversion technologies has received considerable attention recently.

**Fig. 1:**
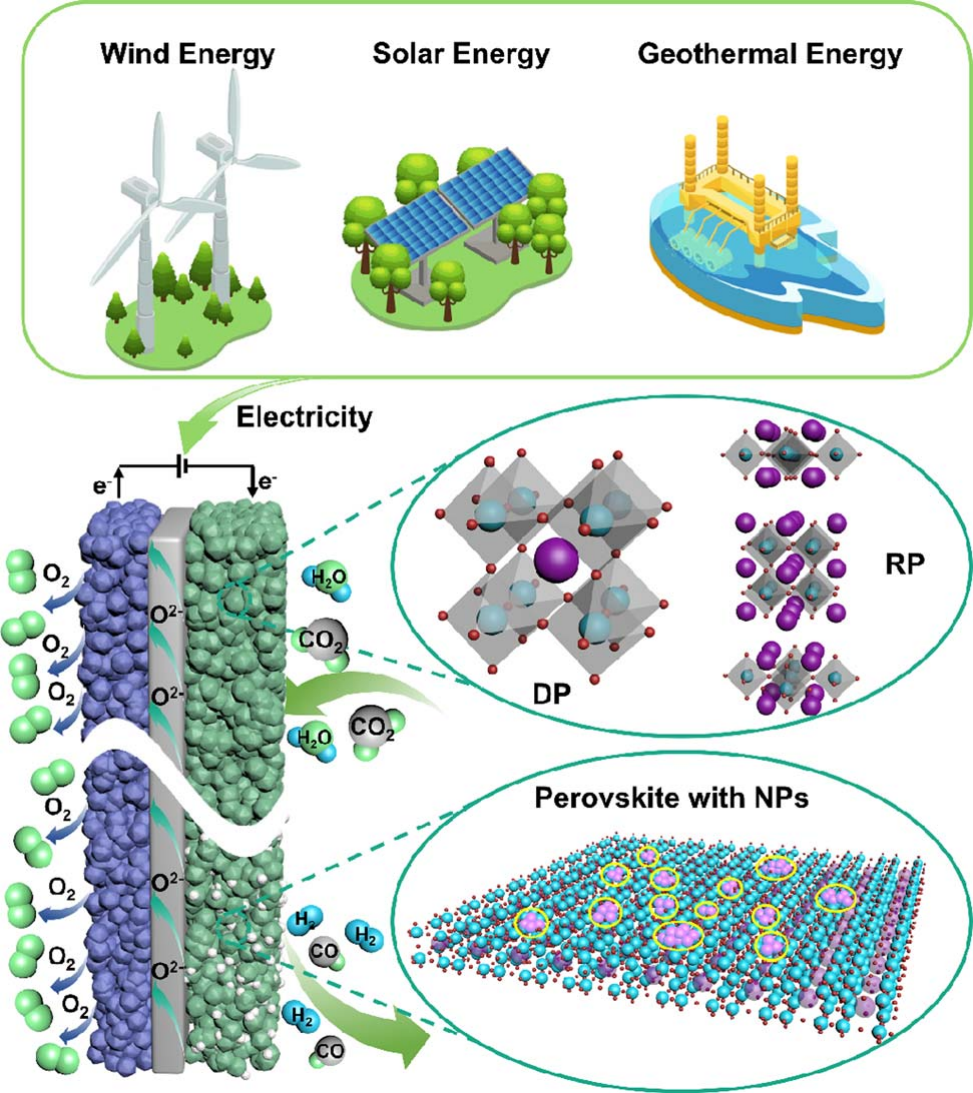
A typical schematic of an SOEC.

Over the past few decades, technologies like carbon capture, utilization and storage (CCUS) and electrochemical conversion have emerged as the most promising pathways to achieving carbon balance. Unlike CCUS, electrochemical conversion methods including solid oxide electrolysis cells (SOECs) (Fig. 1), alkaline electrolysis technology, and proton exchange membrane electrolyzers can efficiently convert CO_2_ into value-added carbon-containing chemicals, likely hydrocarbons, and CO, which are in principle more attractive. Such conversion processes can actually use renewable energy as an energy input for energy storage; thus, CO_2_ can be considered just an intermediate energy carrier in these processes. Among the various available electrochemical technologies, high-temperature CO_2_ electrolysis *via* SOECs offers remarkable advantages, such as high current density, superior stability, and high faradaic efficiency approaching 100%, rendering it more suitable for practical applications.^2–4^ Different from low-temperature electrolysis, the product distribution and selectivity are highly dependent on the electrocatalysts and operation conditions; high-temperature SOECs allow the use of pure CO_2_ as feedstock to produce CO with 100% selectivity, while CO production from CO_2_ is believed to be one of the most economical strategies for CO_2_ conversion.^5^

A SOEC single cell is mainly composed of a porous cathode, a porous anode, and a dense electrolyte. Among these, CO_2_ reduction reactions (CO_2_RR) to form CO, including CO_2_ adsorption and dissociation, and CO desorption, predominantly take place at the cathode. Consequently, the efficiency of SOECs for CO_2_ electrolysis is highly dependent on the electrocatalytic activity of the cathodes (fuel electrodes).^6^ Up to now, nickel-based metal ceramics, such as Ni/(yttrium-stabilized zirconia) YSZ, are still the most commonly used CO_2_RR cathodes in SOECs, exhibiting excellent catalytic activity at high temperatures. However, the stability of the catalytically active sites in nickel-based metal ceramics remains unsatisfactory. Over prolonged operation, the oxidation, aggregation, and carbon deposition of nickel emerge as primary causes for the significant decline in the performance of nickel-based cathodes.^7,8^ Perovskite-type mixed ionic and electronic conductors are considered promising substitutes for nickel-based cathodes because of their good anti-coking ability and excellent stability, but they usually show limited CO_2_RR activity. Hence, numerous studies are focused on enhancing the CO_2_ electrocatalytic activity and stability of perovskite *via* doping, infiltration, *in situ* exsolution, and high-entropy modification strategies. Nonetheless, the stability of current laboratory-grade perovskites fails to meet the demands of industrial-grade stacks, which limits the industrial application, requiring further exploration and optimization. A good summary of the recent progress and challenges in perovskite-type CO_2_RR catalysts for SOECs, in particular the fundamental understanding of the reaction processes and cell deterioration mechanism, will provide useful updated information and guidance to the researchers for their further studies and development. Presently, numerous review articles offer summaries of the material categories, performance comparisons, and the current state of research on SOECs in CO_2_ electrolysis. Nonetheless, there is a lack of a comprehensive overview of methodologies to enhance the properties and electrochemical performance of perovskite-based cathode materials and overlook theoretical-level approaches for SOEC development.

In this review article, we aim to provide an up-to-date summary of the recent progress in the development of non-Ni-YSZ cermet cathodes for the CO_2_RR to CO in SOECs, with particular attention paid to perovskite oxides and their modification. We first summarize the current understanding of the reaction mechanism of CO_2_ electrolysis to CO based on SOEC technology and briefly discuss the current challenges. To tackle the low electronic and ionic conductivity and limited catalytic activity issues of perovskite oxides, we mainly reviewed the modification strategies recently applied to such SOEC cathodes. Additionally, we examined the use of machine learning methods for rapidly identifying promising new perovskite-type cathode materials. Density functional theory (DFT) calculations and advanced *in situ* characterization have recently proven to be powerful tools to deepen the understanding of the CO_2_RR mechanism from both experimental and theoretical perspectives. We also reviewed the recent progress in using these techniques for understanding the CO_2_RR over perovskite-type electrocatalysts at elevated temperatures. Finally, we offer some guidelines for the further development of industrially applicable SOEC cathodes.

## 2. Mechanism of CO_2_ electrolysis over perovskite electrocatalysts in SOECs

Presently, reducing the reaction temperature to enhance operational stability stands as one of the primary challenges in CO_2_ electrolysis within SOECs; however, the mechanisms underlying CO_2_ electrolysis for many materials remain unclear. Consequently, comprehending the reaction mechanisms linked with diverse cathode types is pivotal for crafting cathodes tailored for CO_2_ electrolysis.

The typical setup of SOECs employed for CO_2_ electrolysis is composed of an oxygen ion conductor electrolyte and a porous cathode and a porous anode on both sides.^9,10^ The porous structure of the cathode and anode facilitates gas transportation. The dense electrolyte can isolate the gas on both sides and prevent the direct reaction of the gas inside the cell. In addition, the insulating nature of the electrolyte can prevent cell short circuiting caused by electron transmission.^11^ For a SOEC with oxygen ion conducting electrolyte, CO_2_ is reduced at the cathode to form CO and O^2−^ by acquiring two electrons as shown in eqn (1); subsequently, O^2−^ passes through the electrolyte to the anode side, where it forms O_2_ with the release of electrons, as expressed in eqn (2).

1 CO_2_ (g) + 2e^−^ → CO (g) + O^2−^

2 O^2−^ → 1/2O_2_ (g) + 2e^−^

The C=O in the molecular structure of CO_2_ possesses a high bond energy (approximately 750 kJ mol^−1^), thereby presenting a significant challenge to the catalytic activity of the SOEC cathodes for CO_2_ activation.^12^ In SOECs, the CO_2_RR entails three fundamental processes: CO_2_ adsorption, CO_2_ dissociation, and CO desorption, which are widely acknowledged.

Currently, there are two distinct CO_2_ electroreduction mechanisms based on two different types of cathode materials. In pure perovskite oxides, surface oxygen vacancies 
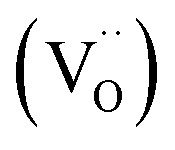
 are believed to be the hosts for CO_2_ chemisorption at high temperatures.^13^ Kozokaro *et al.* employed DFT calculations to conduct an extensive investigation into the CO_2_ electrolysis mechanism on La_0.3_Sr_0.7_Fe_0.7_Cr_0.3_O_3_ (LSFCr) perovskite. Their calculations revealed that CO_2_ is predominantly associated with oxygen ions on the BO_2_ (B = Cr and Fe) plane of LSFCr to form carbonate (CO_3_^2−^) species. Furthermore, the high concentration of oxygen vacancies aids in mitigating the strong adsorption of carbonate, thereby facilitating the activation of CO_2_ molecules and the subsequent desorption of CO.^14^3
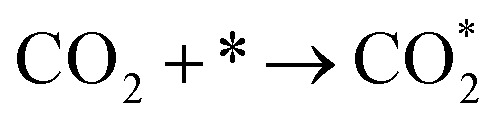
4
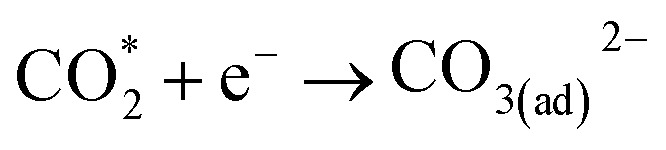

5 CO_3(ad)_^2−^ + e^−^ → CO* + O_(ad)_^2−^

6 CO* → CO

Ricardez-Sandoval *et al.* found that the basicity of surface O^2−^ ions of the (0 0 1) facet of La_0.5_Sr_0.5_FeO_3−*δ*_ perovskite makes it more likely to attract and bind with the C atom of CO_2_ (Lewis acid center) thus forming carbonates.^13^7
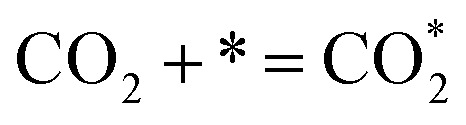
8
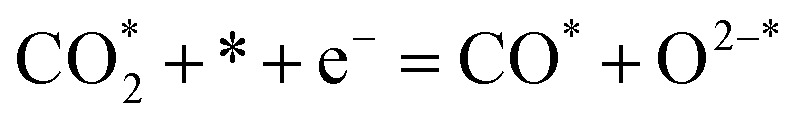

9 CO* = CO + *

Metal–oxide interfaces created through infiltration or exsolution demonstrated superior CO_2_ electrolysis performance compared to pure perovskite oxide interfaces. This is attributed to the enhanced suitability of the active sites at the metal–oxide interface for CO_2_ adsorption and activation.^15,16^10
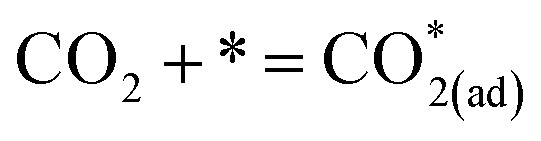
11
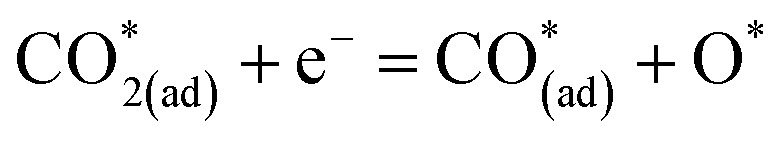
12
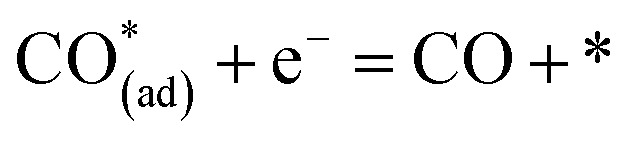


## 3. Advances in perovskite-based CO_2_RR electrocatalysts for SOECs from experimental investigations

**Fig. 2:**
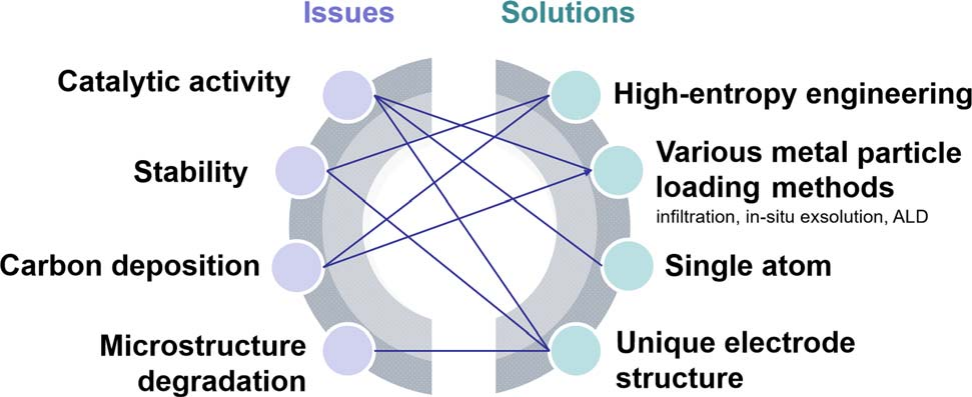
Schematic diagram of the current challenges and their solutions.

Typical perovskite-based oxides consist predominantly of alkaline earth or rare earth metal ions occupying their A lattice site, alongside transition metal ions occupying the B lattice site. This category encompasses simple perovskite structures (ABO_3−*δ*_), Ruddlesden–Popper phases (A_2_BO_4+*δ*_), and double perovskites (AA′B_2_O_5+*δ*_). As mixed ionic–electronic conductivity (MIEC) electrodes, the perovskite may enlarge the active sites towards the entire perovskite surface, compared with the cermet electrode. However, the relatively poor catalytic activity and stability are the main challenges for most perovskite-related oxides, as illustrated in Fig. 2. Strategies towards improving the activity and durability of perovskite CO_2_RR electrocatalysts in SOECs include the introduction of high entropy into the oxide lattice, surface modification with nanoparticles through various methods, or even the introduction of single atom catalysts. By integrating these optimizations with targeted electrode structure design, challenges such as low catalytic activity, poor stability, and carbon deposition in perovskite-based electrode materials are effectively mitigated.

### 3.1 High entropy perovskite oxides

The field of high-entropy materials (HEM) has been gaining significant attention since Yeh and Cantor reported their groundbreaking research on metallic alloys.^17,18^ The homogeneous blending of various elements elevates the system's entropy and yields a structure that is stabilized both thermodynamically (Δ*G* = Δ*H* − T*Δ*S*) and kinetically (slow diffusion), enabling it to endure demanding conditions like high temperatures, corrosion, and high electrochemical potential.^19^ These properties have also been confirmed in other materials with high entropy. Lately, many classes of non-metallic oxides have been added to the category of HEM.^20^ Therefore, the high-entropy doping strategy is expected to solve the problem of electrochemical activity and high-temperature stability that are difficult to realize simultaneously in conventional perovskites.

**Fig. 3:**
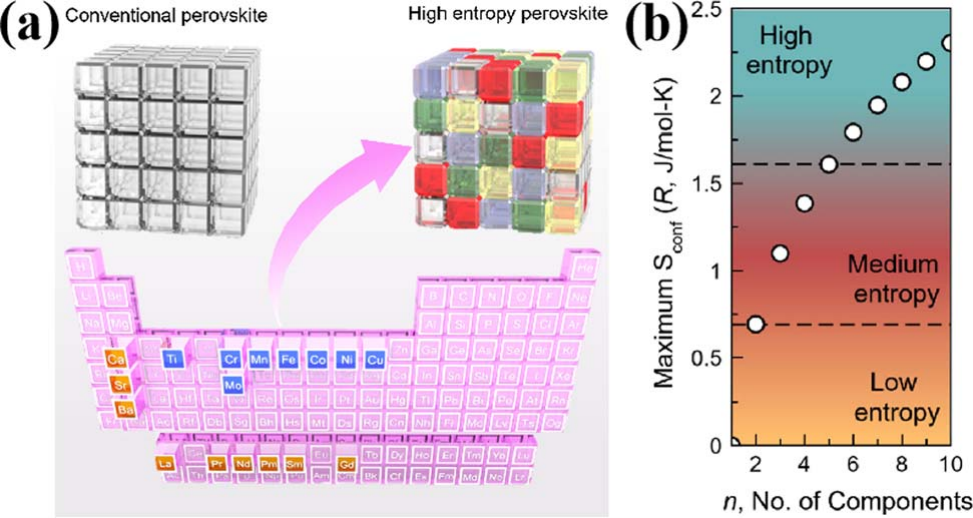
(a) Typical schematic of a high-entropy perovskite. (b) Ideal configurational entropy as a function of the number of elements, *n*, in equiatomic proportions. Reproduced from ref. 21. Copyright 2019, The American Chemical Society.

As depicted in Fig. 3a, the presence of multiple elements (four, five, or more) occupying the same crystallographic site could result in considerable configurational disorder in HEM. The definition of high-entropy perovskite asymptotically follows the concept of high-entropy alloys. Nonetheless, there isn't a single, widely accepted definition of what constitutes a high-entropy substance. There are two commonly accepted definitions: (1) configurational entropy larger than 1.6*R* (Fig. 3b), and (2) single phase and equimolar.^22^ Many investigations have evaluated the perovskite structure by mixed entropy (Δ*S*_mix_), Goldschmidt tolerance factor (*t*), and A/B site cation size difference (*δ*).^21,23–25^ The molar configurational entropy of the perovskite oxide can be calculated according to eqn (13):13
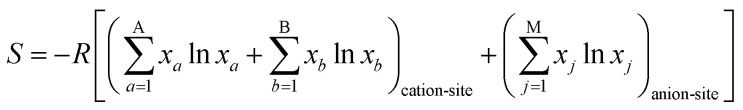
where *x*_*a*_, *x*_*b*_ and *x*_*j*_ are the mole fractions of elements found in the cation and anion sites, respectively, with *R* being the universal gas constant. The Goldschmidt tolerance factor (*t*) is employed for evaluating the perovskite structure.14
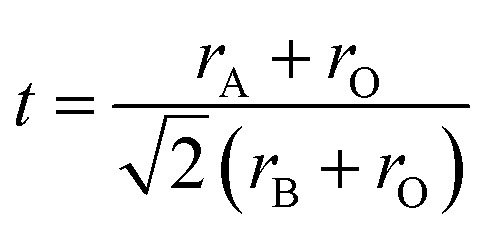
where r_A_ and *r*_B_ are the ionic radii of the cations on the A and B sites, respectively, and *r*_O_ is the radius of the oxygen ion. For an ideal cubic perovskite structure, *t* is equal to 1. Research indicates that the *t* value for a stable perovskite structure is expected to be in the range of 0.78 < *t* < 1.05.15
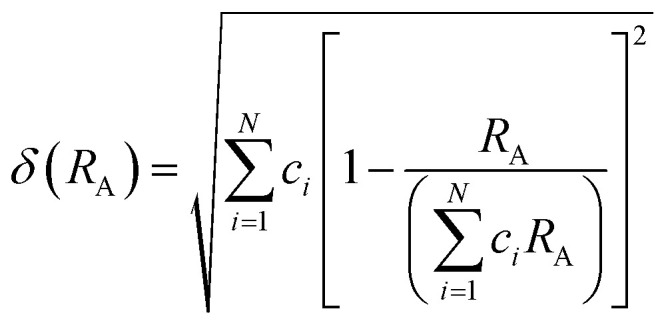
where *R*_A_ is the radius of the *i*th cation on the A site, and *c*_*i*_ is the mole fraction of the *i*th cation. The observed *δ*(*R*_A_) values in the HEPOs varied from 9.9 to 13%, which is in line with earlier research and within the boundaries set by the Hume-Rothery solid-solution rule.^24,26^Table 1 lists several typical high-entropy perovskite oxides that have been utilized as electrodes for SOFCs/SOECs. HEM primarily consist of high-entropy perovskites and spinels. Present research predominantly centers on the air electrode of SOFCs, and related investigations concerning the utilization of HEM for CO_2_ electrolysis in SOECs have emerged. Depending on the doping position, HEM can be categorized into A-site high entropy and B-site high entropy. Despite numerous high-entropy perovskites being documented, the elements chosen for the A-site or B-site are often similar.

**Table 1:** Recent applications of HEM in solid oxide cells

Applications	Materials	Ref.
R-SOEC (CO_2_)	Sr_2_(Fe_1.0_Ti_0.25_Cr_0.25_Mn_0.25_Mo_0.25_)O_6−*δ*_	27
SOEC cathode (CO_2_)	Sr_2_FeCo_0.2_Ni_0.2_Mn_0.1_Mo_0.5_O_6−*δ*_	28
SOEC cathode (CO_2_)	Pr_0.5_Ba_0.5_Mn_0.2_Fe_0.2_Co_0.2_Ni_0.2_Cu_0.2_O_3−*δ*_	29
SOEC cathode (CO_2_)	Pr_0.8_Sr_1.2_(CuFe)_0.4_Mo_0.2_Mn_0.2_Nb_0.2_O_4−*δ*_	30
R-SOEC (H_2_O)	Pr_0.2_Ba_0.2_Sr_0.2_La_0.2_Ca_0.2_CoO_3−*δ*_	31
SOFC air electrode	La_0.2_Pr_0.2_Nd_0.2_Sm_0.2_Sr_0.2_CoO_3−*δ*_	32
SOFC air electrode	(La_0.2_Nd_0.2_Sm_0.2_Ca_0.2_Sr_0.2_)MnO_3_	25
SOFC air electrode	(La_0.2_Pr_0.2_Nd_0.2_Sm_0.2_Gd_0.2_)BaCo_2_O_5+*δ*_	33
SOFC air electrode	(La_0.2_Pr_0.2_Sm_0.2_Gd_0.2_Nd_0.2_)Ba_0.5_Sr_0.5_Co_1.5_Fe_0.5_O_5_	34
SOFC air electrode	(La_0.2_Sr_0.2_Pr_0.2_Y_0.2_Ba_0.2_)Co_0.2_Fe_0.8_O_3−*δ*_	35
SOFC air electrode	(La_0.2_Pr_0.2_Nd_0.2_Sm_0.2_Gd_0.2_)_2_CuO_4_	36
SOFC air electrode	(Fe_0.2_Mn_0.2_Co_0.2_Ni_0.2_Zn_0.2_)_3_O_4_	37
SOFC air electrode	La_0.7_Sr_0.3_(Co,Cr,Fe,Mn,Ni)O_3−*δ*_	38
SOFC air electrode	LaMn_0.2_Fe_0.2_Co_0.2_Ni_0.2_Cu_0.2_O_3−*δ*_	39
SOFC air electrode	Ni(Fe_0.2_Mn_0.2_Co_0.2_Cr_0.2_Ni_0.2_)_2_O_4_	40
SOFC air electrode	La_0.7_Sr_0.3_Co_0.25_Fe_0.25_Ni_0.25_Mn_0.25_O_3−*δ*_	41
SOFC air electrode	La_0.2_Pr_0.2_Nd_0.2_Sm_0.2_Ba_0.1_Sr_0.1_Co_0.2_Fe_0.6_Ni_0.1_Cu_0.1_O_3−*δ*_	42
SOFC anode	FeCoNiCuAl	43
SOFC electrolyte	BaCe_0.4_Zr_0.4_Y_0.15_Ni_0.01_Cu_0.01_Co_0.01_Fe_0.01_Zn_0.01_O_3_	44
PCFC electrolyte	BaSn_0.16_Zr_0.24_Ce_0.35_Y_0.1_Yb_0.1_Dy_0.05_O_3−*δ*_	45
R-PCEC	Pr_1/6_La_1/6_Nd_1/6_Ba_1/6_Sr_1/6_Ca_1/6_CoO_3−*δ*_	46
R-PCEC	BaHf_1/6_Sn_1/6_Zr_1/6_Ce_1/6_Y_1/6_Yb_1/6_O_3−*δ*_	24
PCFC air electrode	La_1.2_Sr_0.8_Mn_0.2_Fe_0.2_Co_0.2_Ni_0.2_Cu_0.2_O_4+*δ*_	47
PCFC air electrode	Fe_0.6_Mn_0.6_Co_0.6_Ni_0.6_Cr_0.6_O_4_	48
PCFC air electrode	BaCo_0.2_Zn_0.2_Ga_0.2_Zr_0.2_Y_0.2_O_3−*δ*_	49

The A-site high-entropy perovskite comprises at least five near-equimolar metal cations with similar ionic radii (La^3+^, Pr^3+^, Nd^3+^, Pm^3+^, Sm^3+^, Gd^3+^, Ba^2+^, Sr^2+^, Ca^2+^), thereby minimizing lattice mismatch. Meanwhile, a “cocktail” effect takes place as a result of the crystal lattice distortion and component mixing, wherein the characteristics of the final material differ dramatically from the properties of the individual components. A-site cations can affect the overall catalytic activity of the perovskite by influencing the oxygen vacancy concentration of the transition metal at the B-site, the metal–oxygen bond strength, oxidation, and spin states.^50^ Up to now, only a few A-site HEM have been utilized as cathodes in SOECs to investigate their performance in CO_2_ electrolysis. For example, it was reported that the lattice distortion caused by the highly dispersed A-site ions in La_0.2_Pr_0.2_Nd_0.2_Sm_0.2_Sr_0.2_MnO_3−*δ*_ (HE-LSM) effectively inhibited Sr segregation at high temperatures,^51^ and the distinctive high-entropy structure enabled Ni-YSZ|YSZ|GDC|HE-LSM single cells to exhibit robust stability during long-term operation. This fully demonstrates that the concept of high entropy in the A-site provides a novel perspective for designing a stable cathode of SOFCs. In addition to a variety of alkaline-earth and rare-earth metal doping, alkali metal multiple doping is worth exploring.^52,53^

**Fig. 4:**
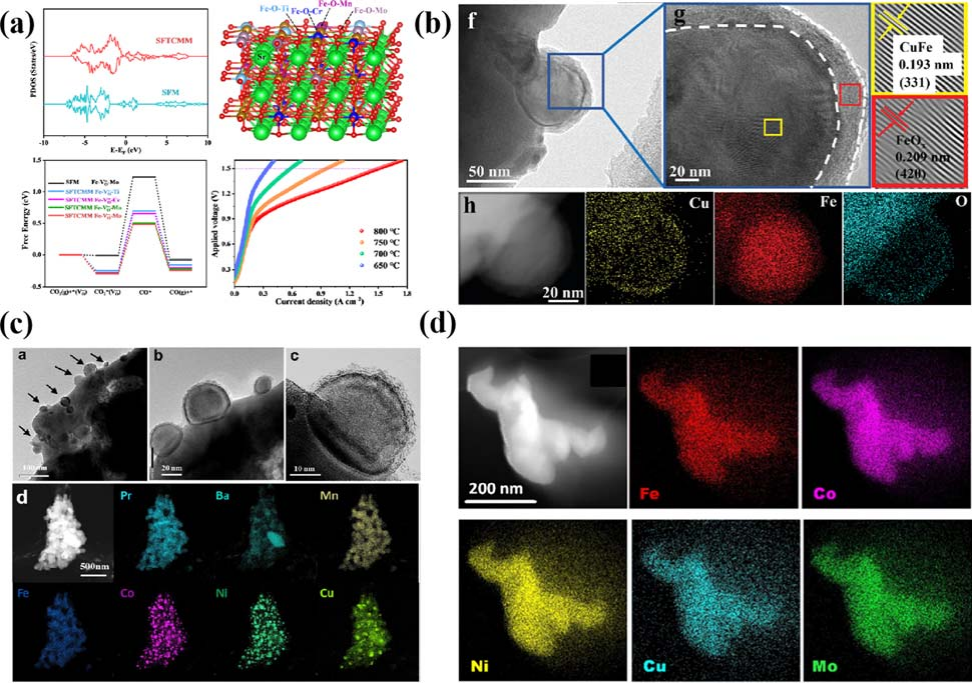
(a) Theoretical calculations and *I*–*V* curves for CO_2_ reduction of SFTCMM and SFM. Reproduced from ref. 27. Copyright 2023, The American Chemical Society. (b) TEM, HRTEM, and the corresponding EDX mapping of PSCFMMN after reduction in H_2_ at 800 °C for 2 h. Reproduced from ref. 30. Copyright 2023, Wiley-VCH. (c) HRTEM, HAADF-STEM, and EDS element mappings of reduced HE-PBM with exsolved NPs. Reproduced from ref. 29. Copyright 2023, Elsevier. (d) STEM-EDS image of the five elements in the alloy. Reproduced from ref. 55. Copyright 2024, The American Chemical Society.

The B-site high entropy exhibits significant potential in the design and optimization of the perovskite by exploiting the distinct effects of each element on electrochemical properties. High-entropy alloys (HEAs) reside in a state of near non-equilibrium thermodynamics due to significant lattice distortion resulting from atomic size mismatches. As a result, during the catalytic process, HEAs have a lower energy barrier due to their larger potential energy.^54^ Particularly, when integrated with an exsolution strategy, the incorporation of multiple elements into the B site of perovskite oxides can enable the exsolution of multimetallic or alloy NPs driven by hydrogen reduction or an applied electric potential. He *et al.* introduced a novel medium-entropy perovskite, Sr_2_(Fe_1.0_Ti_0.25_Cr_0.25_Mn_0.25_Mo_0.25_)O_6−*δ*_ (SFTCMM), aiming to augment CO_2_ activation by bolstering metal 3d–O 2p hybridization and diminishing the O 2p bond center (Fig. 4a).^27^ The SFTCMM-based symmetrical SOECs delivered 1.50 A cm^−2^ at 800 °C and 1.5 V. Yang *et al.* reported a new layered perovskite, Pr_0.8_Sr_1.2_(CuFe)_0.4_Mo_0.2_Mn_0.2_Nb_0.2_O_4−*δ*_ (HE-PSCFMMN).^30^ Through the reduction of PSCFMMN, an *in situ* exsolution of the CuFe alloy@FeO_*x*_ (CFA@FeO) core–shell structure was achieved (Fig. 4b). Unlike simple *in situ* exsolved NPs, the abundance of oxygen vacancies in the FeO_*x*_ shell permitted the triple phase boundary (TPB) to extend from the CFA@FeO/HE-PSCFMMN interface to the whole FeO_*x*_ shell. *In situ* exsolved Fe–Co–Ni–Cu quaternary alloy nanocatalysts from a Pr_0.5_Ba_0.5_Mn_0.2_Fe_0.2_Co_0.2_Ni_0.2_Cu_0.2_O_3−*δ*_ (HE-PBM) cathode exhibited excellent electrochemical performance and stability (Fig. 4c).^29^ Recently, a study has reported an Fe_0.1_Co_0.35_Ni_0.35_Cu_0.1_Mo_0.1_ (FCNCM) quinary HEA as a cathode in SOECs (Fig. 4d).^55^ Due to larger particle sizes, predominantly having a magnitude of several tens of micrometers, the high-entropy fuel electrode exhibited a reduced specific surface area compared to the perovskite electrode. Moreover, after the stability test, slight oxidation of the alloy occurred. Oxidation would lower the electronic conductivity and potentially trigger the mechanical strength to degrade.

High-entropy perovskite oxides (HEOs) have emerged as promising materials for fuel electrodes in the electrochemical reduction of CO_2_. The incorporation of multiple elements into HEOs serves to stabilize the lattice *via* configurational entropy, thereby preserving the original perovskite structure while concurrently enhancing catalytic activity. Moreover, the integration of HEAs with perovskite-based materials can be achieved through various techniques such as mixing and infiltration to develop exceptional cathodes. The HEA formed by the combination of the B-site high entropy and *in situ* exsolution strategy is expected to exhibit an efficient and robust cathode of SOECs. Despite this, the precise impact of entropy alone on the catalytic activity enhancement of B-site high-entropy perovskites remains uncertain. Traditional high-entropy alloys have not received the same level of scrutiny as high-entropy perovskites. It is imperative to conduct comprehensive studies to enhance our understanding and achieve significant short-range disorder in HEAs, thereby unlocking their full potential. Moreover, the inherent complexity of multi-elemental synthesis presents a synthesis challenge, compounded by the ease of phase separation and elemental segregation among multiple distinct elements.^19^ In conclusion, additional research is necessary to elucidate the distinctive band structure, identify catalytic sites, and explore the *in situ* formation of advantageous phases in HEAs.

### 3.2 Surface modification with single atom catalysts

As early as the 1960s, Boudart *et al.* discovered, through hydrogen adsorption isotherms, that the H/Pt ratio on extremely low-loading platinum catalysts was very close to 1 : 1, thus demonstrating the possibility of Pt existing in the form of single atoms.^56^ In the 1990s, Nellist *et al.* employed transmission electron microscopy (TEM) technology to distinctly observe platinum and rhodium dispersed as single atoms on γ-Al_2_O_3_ for the first time.^57^ Subsequently, in 2011, Zhang *et al.* synthesized single atom catalysts (SACs) comprising individual Pt atoms dispersed on the surface of iron oxide nanocrystals (Pt_1_/FeO_*x*_) and utilized them in heterogeneous catalysis for the first time. The Pt_1_/FeO_*x*_ SACs demonstrated superior stability, high activity, and preferred oxidation of CO in H_2_, along with an unmatched atom use efficiency.^58^ In recent years, the introduction of SACs has garnered increasing research attention. Due to their exceptional atom utilization and diverse catalytic activities and selectivities, SACs find wide applications in the hydrogen evolution reaction (HER), oxygen evolution reaction (OER), oxygen reduction reaction (ORR), carbon dioxide reduction reaction (CO_2_RR), nitrogen reduction reaction (NRR), and other rapidly advancing fields. In contrast to traditional NPs and nanocluster catalysts, single atom structures offer enhanced precision and flexibility in adjusting the crystal, coordination, and electronic structure of the catalyst. Through minimal precious metal loading, SACs can achieve outstanding catalytic performance, presenting a resource-efficient and effective approach. The synthesis of SACs stands as a research priority. With the continuous emergence of advanced catalyst synthesis methods, there has been a concomitant enhancement in the catalytic performance of catalysts. Nonetheless, the precise manipulation of atoms in the synthesis of theoretically designed SACs remains a formidable challenge. Driven by ultra-high surface free energy, individual atoms have a propensity to readily migrate and aggregate into NPs.

**Fig. 5:**
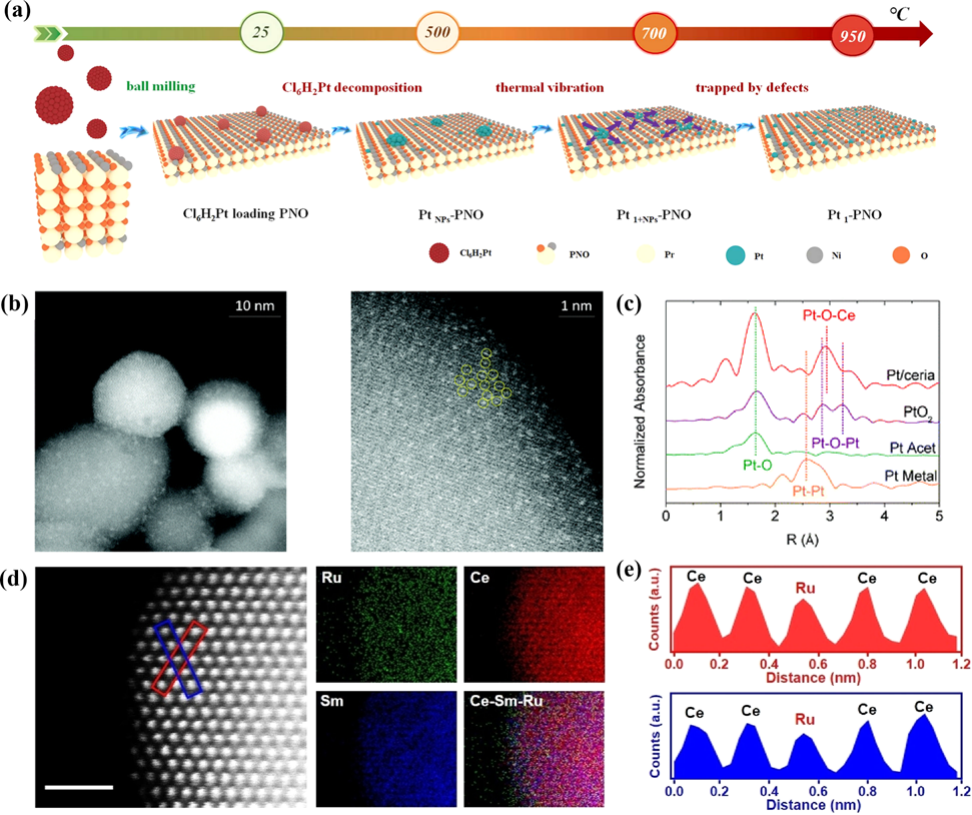
(a) Schematic image of Pt-loaded onto PNO to form Pt1–PNO. Reprinted with permission from ref. 60. Copyright 2022, Elsevier. (b) Probing atomic Pt: HAADF-STEM images of Pt/ceria. (c) *k*^2^-Weighted Fourier transform of the Pt L3-edge EXAFS spectra of Pt/ceria and model compounds. Reprinted with permission from ref. 61. Copyright 2022, Elsevier. (d) Atomic-scale HAADF-STEM images and EDS elemental maps of SDCRu-1100 and (e) the corresponding intensity profiles are labeled with the blue boxes in (d). Reproduced from ref. 62. Copyright 2023, Wiley-VCH.

Apart from their extensive utilization in low-temperature catalysis, SACs have recently begun to find gradual application in high-temperature solid oxide cells (SOCs) over the last two years. Inspired by the atomic trapping mechanism, Li *et al.* introduced a reverse atom trapping strategy.^59^ At high temperature, MoO_3_ was employed to trap Sr atoms on the surface of (La_0.6_Sr_0.4_)_0.95_Co_0.2_Fe_0.8_O_3−*δ*_ (LSCF), leading to the creation of additional Sr/O vacancies and higher covalency of (Co/Fe)–O bonds, as well as some harmless SrMoO_4_ phases. Compared with the pristine LSCF cathode, the oxygen reduction activity of Sr_vac_/LSCF was significantly enhanced. Within the temperature range of 873–973 K, the cell performance using Sr_vac_/LSCF-1% as the cathode exhibited an improvement of over 70%. Unlike the conventional method of controlling oxygen vacancy concentration in perovskites through A/B site doping, reverse atomic trapping achieved the activation of lattice oxygen in perovskites with atomic precision. Li *et al.* proposed a custom method to selectively anchor Pt atoms to the B-site in Pr_4_Ni_3_O_10+*δ*_ using a simple and universally applicable mechanical mixing approach. This single-atom catalyst not only withstood treatment at 700 °C for 800 hours in air but also saw its electrochemical performance improve by nearly 100%.^60^ Shin *et al.* reported a single-atom Pt/ceria nanocatalyst loaded in the oxide fuel electrode of a SOC *via* an *in situ* synthetic process, which can securely anchor isolated Pt atoms on the surface of ceria NPs and suppress their high-temperature migration.^61^ Wang *et al.* recently synthesized SACs (SDCRu-1100) where a solitary ruthenium (Ru) atom was anchored on the surface of an oxygen ion conductor, Ce_0.8_Sm_0.2_O_2−*δ*_ (SDC), exploiting the phenomenon that robust covalent metal–support interactions under certain conditions can securely anchor a single atom (Fig. 5d and e).^62^ This catalyst found application in the direct electrolysis of CO_2_ in SOECs. Due to the distinctive coordination environment and electronic structure of SDCRu-1100, the existence of single atom Ru enhanced the oxygen hole concentration and conductivity of SDC. Compared to the pristine SDC, DFT calculations revealed that SDCRu-1100 exhibited robust CO_2_ adsorption and CO desorption capabilities. These findings collectively signify that SDCRu-1100 served as a highly efficient catalyst for the electrolysis of CO_2_ in SOECs. SDC and SDCRu-1100 were mixed with La_0.6_Sr_0.4_Co_0.2_Fe_0.8_O_3−*δ*_ (LSCF) to fabricate composite SDC–LSCF and Ru_1_/SDC–LSCF cathodes for electrochemical performance evaluation. In contrast to SDC–LSCF, the current density of the Ru_1_/SDC–LSCF cathode reached 1.68 A cm^−2^ at 1.6 V, representing a notable enhancement of 54.13%.

The integration of SACs into perovskite materials has demonstrated potential for enhancing the performance of SOECs. However, extensive studies have demonstrated that NPs on electrode surfaces tend to agglomerate during high-temperature operation, leading to performance degradation. The interactions between perovskite carriers and single atoms, as well as the stability of single-atom active sites within electrodes under operational conditions, remain underexplored. In contrast, the field of low-temperature catalysis has made significant progress in understanding the stabilization mechanisms of single atoms and their coordination environments with supports. For instance, studies on catalysts using metal–organic frameworks (MOFs) as single-atom carriers have shown that various amine sites within MOFs can form strong metal–nitrogen bonds with metal atoms, thereby stabilizing these atoms during high-temperature pyrolysis preparation.^63,64^ Yao *et al.* confirmed the capture and anchoring effects of defects in graphene on Ni atoms through advanced characterization techniques such as high-angle annular dark-field scanning transmission electron microscopy (HAADF-STEM).^65^ However, current research on SACs in high-temperature applications remains superficial, lacking necessary studies on the coordination of single atoms with perovskite surfaces. Therefore, future research on SACs for SOECs should focus on the state of single atoms within the catalyst. This will provide a deeper understanding of their stability during long-term operation, facilitating widespread commercialization of SOECs.

### 3.3 Surface modification with nanoparticles

Since the perovskite oxide in SOECs is well sintered with limited surface area, and the surface properties of the perovskite are sometimes difficult to tailor, the deposition of the perovskite surface with some NPs was found to be an effective way to increase the surface active surfaces and to tailor the surface adsorption capability and other properties, to adjust the catalytic performance of the perovskite electrocatalyst for the CO_2_RR in SOECs. There are several ways to introduce the NPs onto the perovskite surface, including infiltration and exsolution, and each method has its advantages and disadvantages.

#### 3.3.1 Infiltration

Infiltration is a simple method to introduce NPs on the surface of the perovskite scaffold, which can effectively increase CO_2_RR active sites in SOEC cathodes. By infiltrating catalytically active species onto the perovskite support, it is possible to enhance the chemical adsorption of CO_2_ and foster the formation of active carbonate intermediates. Given that only low-temperature calcination (<800 °C) is necessary during the infiltration process, excessive growth and agglomeration of the infiltrated particles are avoided.^66^ Directly infiltrating metal catalysts (Co, Fe, Ni, and Ru) as active species on the surface of La_0.6_Sr_0.4_Fe_0.9_Mn_0.1_O_3_ (LSFM) can significantly promote the CO_2_RR activity of the cathodes.^67^ Research comparisons indicated that under a 50% CO/CO_2_ atmosphere, the Fe particle-infiltrated LSFM cathode demonstrated the most optimal electrolytic performance, achieving an electrolysis performance of 2.20 A cm^−2^ at 1.5 V and 850 °C. CeO_2_ is an outstanding mixed ion/electron conductor. The abundance of the reversible redox pair Ce^3+^/Ce^4+^ endowed it with remarkable anti-carbon deposition properties during the catalytic conversion of carbon-containing molecules.^68^ La_0.3_Sr_0.7_Ti_0.3_Fe_0.7_O_3−*δ*_-CeO_2_ (LSTF0.7-CeO_2_) was prepared by using the infiltration method as a fuel electrode for the 50% CO/CO_2_ conversion reaction in a reversible SOC (LSTF0.7-CeO_2_|ScSZ|LSM-ScSZ).^66^ In SOEC mode, the current densities reached 2.36 A cm^−2^ at 2.0 V and 800 °C. The composite cathode SFM-SDC is a promising material for SOECs, enhancing CO_2_RR efficiency through increased active sites and TPBs compared to the SFM cathode. The infiltrated electrode can further increase the electrode TPB area compared to the composite electrode, offering an effective method for constructing highly active electrode materials. Lv *et al.* infiltrated Gd_0.2_Ce_0.8_O_1.9_ (GDC) NPs onto the Sr_2_Fe_1.5_Mo_0.5_O_6−*δ*_ (SFM) surface enhancing the oxide ionic conductivity of the SFM electrode, leading to improved CO_2_RR efficiency.^69^ The current density reaches 0.446 A cm^−2^ at 1.6 V and 800 °C. Improving the CO_2_ adsorption capacity on the perovskite oxide surface enhances the generation of active carbon intermediates, thereby significantly enhancing electrochemical performance. Zhang *et al.* show that different alkaline-earth metal compounds were infiltrated into SFM perovskite oxides as cathodes enhancing the chemisorption and activation capacity of CO_2_.^70^ Moreover, the co-infiltration of Pd and GDC NPs onto the LSCM cathode significantly enhanced the electrocatalytic activity and anti-coking properties.^71^ The extensively distributed Pd–GDC NPs improved the electrochemical performance of LSCM by increasing the number of active reaction sites and forming many metal/oxide interfaces.

Loading NPs on the surface of perovskites to enhance the electrochemical performance of electrodes is a well-established and effective methodology. Through a simple immersion process that infiltrates precursor solutions into the electrode scaffold, nanostructured catalyst composite electrodes can be fabricated. This approach leads to the enhancement of the triple phase boundary (TPB), thereby effectively improving the electrochemical performance. Due to the weak NP–substrate interaction and high surface energy of the NPs of the electrode prepared by the infiltration method, at high operating temperatures, agglomeration of NPs remains a major problem leading to performance degradation of the cathode.^72^ Improving the substrate–NP interaction is key to further enhancing the operational stability of such NP modified perovskite electrodes in high-temperature CO_2_RR.

#### 3.3.2 *In situ* exsolution

An alternative method to infiltration for introducing NPs onto the perovskite surface is the *in situ* exsolution method. Through the incorporation of reducible metals such as Fe, Co, Ni, and Cu into the perovskite oxide, coupled with high-temperature reduction treatment, these elements can undergo migration from the bulk to the surface of the perovskite, ultimately coalescing to form metal NPs. Unlike precipitating NPs onto the perovskite surface, exsolved analogs become tightly embedded within the host perovskite structure, thereby effectively mitigating the tendency for NP agglomeration.^73^ Following reduction at 800 °C, the *in situ* exsolved Ni NPs derived from (La_0.75_Sr_0.25_)_0.9_(Cr_0.5_Mn_0.5_)_0.9_Ni_0.1_O_3−*δ*_ markedly elevated the electrochemical performance, with current density reaching 0.38 A cm^−2^ at 2.0 V, and substantially augmented the faradaic efficiency from 50% to 80% during CO_2_ electrolysis.^74^

**Fig. 6:**
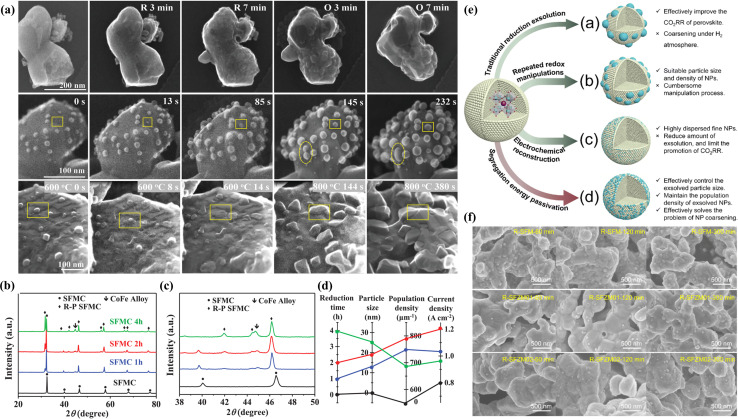
(a) *In situ* STEM images before reduction and after reduction and re-oxidation. (b) XRD patterns under different conditions and (c) the enlarged XRD patterns of (b). (d) The relationship between reduction time, particle size, population density, and current density. Reproduced from ref. 79. Copyright 2020, Wiley-VCH. (e) Design scheme of NPs exsolved from perovskites with different methods. (f) SEM images under different reduction times. Reproduced from ref. 83. Copyright 2023, The Royal Society of Chemistry.

Numerous studies have demonstrated that prolonged annealing in a hydrogen atmosphere induced structural evolution in perovskites. *In situ* exsolved Fe NPs incorporated onto the La_1.2_Sr_0.8_Mn_0.4_Fe_0.6_O_4−*α*_ surface originated from the phase transition of La_0.6_Sr_0.4_Mn_0.2_Fe_0.8_O_3−*δ*_ induced by treating in a H_2_ atmosphere at 800 °C for 10 h. This material displayed noteworthy current densities of 2.04, 1.43, and 0.88 A cm^−2^ at 850, 800, and 750 °C, respectively, at a voltage of 1.5 V.^75^ To further augment the catalytic activity of perovskite electrodes, the design of bimetals (Fe–Cu^76^), alloys (NiFe,^77^ RuFe,^78^ CoFe,^79^ IrFe,^80^*etc.*) and core–shell (NiFe@FeO_*x*_)^81^ NPs on the perovskite surface is becoming increasingly prevalent. For example, Wang *et al.* systematically investigated the CoFe alloy exsolved from the La_0.4_Sr_0.6_Co_0.2_Fe_0.7_Mo_0.1_O_3−*δ*_ (LSCFM) using *in situ* STEM and DFT calculations at the atomic scale. Compared with the LSCFM cathode, CoFe@LSCFM demonstrated an approximately 1.21 times increase in CO_2_ electrolysis performance. Through DFT calculations, this notable enhancement primarily was found to have stemmed from the robust CO_2_ adsorption and dissociation capabilities, facilitated by the highly active metal–oxide interface between CoFe and LSCFM.^15^ In addition to the exploration of diverse exsolved metals, the size and distribution of the exsolved particles are also critical factors influencing catalytic activity. To optimize the metal/oxide interfaces, Wang *et al.* proposed a strategy of repeated redox manipulations to enhance the exsolution of RuFe alloy NPs on the surface of Sr_2_Fe_1.4_Ru_0.1_Mo_0.5_O_6−*δ*_ (SFRuM) perovskite.^78^ This strategy aims to enrich the Ru beneath the perovskite surface, facilitating the exsolution process. After repeated redox manipulations, a substantial number of RuFe particles (∼21 000 μm^−2^) exsolved on the surface of SFRuM, marking a 3.6 times increase compared to the exsolution observed during the first reduction manipulations. The abundant metal/oxide interfaces formed after the repeated redox processes significantly enhanced the CO_2_RR, thereby bolstering the electrochemical performance of the SOECs employing SFRuM-GDC as the cathode. In comparison to the cathode before the reduction, the electrochemical performance of the SOECs after six repeated redox manipulations exhibited an 86.4% increase at 800 °C and 1.2 V. It is noteworthy that repeated redox manipulations can facilitate exsolution while preserving the Fe/Ru ratio and size of the exsolved metal NPs. Neagu *et al.* meticulously investigated the Ni NPs exsolved from La_0.5_Ca_0.4_Ni_0.2_Ti_0.8_O_3−*γ*_.^82^ Through detailed analyses encompassing XRD and SEM images, they discerned that neither the reducing gas nor the reduction time was a decisive factor for exsolution control. Instead, the reduction temperature emerged as the primary determinant, with the particle population decreasing and the particle size increasing as the reduction temperature increased. Upon employing the Ni-exsolved La_0.5_Ca_0.4_N_i0.2_Ti_0.8_O_3−*γ*_ in methane conversion, it became evident that the particle characteristics, encompassing size and population, played a pivotal role in the methane activation temperature, selectivity, and anti-coking properties of the NPs. Moreover, the continual enlargement of NPs in a reducing atmosphere led to particle agglomeration, representing one of the principal causes of the reduction in catalytic activity. As shown in Fig. 6a, Wang *et al.* utilized *in situ* TEM technology to discover the emergence, growth, and agglomeration process of CoFe alloy on the surface of Sr_2_Fe_1.35_Mo_0.45_Co_0.2_O_6−*δ*_ under an H_2_ atmosphere.^79^ They also investigated the connection between the electrochemical performance of SOECs and reduction time, density, and particle size (Fig. 6b–d). Their investigation confirmed that the size of NPs exsolved after prolonged reduction treatment (4 hours) was larger, but the CoFe alloy particle density experienced a sharp decrease, and the electrochemical performance was only three-quarters that achieved with a reduction time of 2 hours.

To address the issue of aggregation of exsolved particles arising from oversized particles in a hydrogen atmosphere, we introduced, for the first time in our previous work, a strategy to selectively regulate the exsolution energy of active metals at the B-site of perovskites, aiming for simple, efficient, and precise control of NPs (Fig. 6e).^83^ By incorporating an appropriate amount of Zn element into Sr_2_Fe_1.5_Mo_0.5_O_6−*δ*_ (SFM), the exsolution energy of Fe from the initial −1.66 eV increased to −1.56 eV, thereby controlling the passivation exsolution of Fe metal and effectively preventing overgrowth and agglomeration of exsolved particles on porous SFM electrodes (Fig. 6f). In comparison to the SFM electrode, the electrolytic cell utilizing Sr_2_Fe_1.4_Zn_0.1_Mo_0.5_O_6−*δ*_ as the fuel electrode exhibited a current density of 2.74 A cm^−2^ at 850 °C and 1.6 V, demonstrating an improvement of approximately 93% in performance.

In addition to hydrogen-driven exsolution, the electrochemical technique of voltage-driven exsolution was employed to construct a more robust metal/oxide interface.^84^ Owing to the sluggish ion diffusion rate in perovskites, the hydrogen-driven exsolution process required several hours to complete. In contrast, the voltage-driven exsolution behavior can typically be completed within a few seconds. Wang *et al.* conducted a comprehensive investigation into the exsolution of IrFe alloy in Sr_2_Fe_1.45_Ir_0.05_Mo_0.5_O_6−*δ*_ (SFIrM) under voltage-driven conditions. The investigation revealed a close correlation between the activation process (*i.e.* exsolution) of the electrode and the applied voltage. Continuous application of a 1.0 V voltage for 4000 s resulted in the gradual stabilization of the current density of the electrolytic cell, indicative of electrode activation completion. At this juncture, particles exsolved on the surface of SFIrM measured approximately 1 nm, with a density of 80 000 μm^−2^. With increasing applied voltage, the duration of electrode activation is significantly shortened (250 s at 1.2 V, 40 s at 1.4 V, 20 s at 1.6 V). Intriguingly, variation in applied voltages does not affect the final density and average particle size of particles on SFIrM. Relative to the SFM-based electrolytic cell, the current density of the IrFe@SFIrM-based electrolytic cell increased by 25.8% at 800 °C and 1.6 V (1.46 A cm^−2^*vs.* 1.16 A cm^−2^), owing to the heightened activity and density modification of IrFe alloy particles.^80^ Current research indicates that the primary hindrance to the practical implementation of voltage-driven exsolution is the limited stability of perovskites under high voltages.^85^ Despite the thermal stability of exsolved NPs being indeed bolstered by certain methods mentioned above, numerous studies have demonstrated that prolonged annealing in a hydrogen atmosphere induced structural evolution in perovskites, thereby affecting the stability of exsolution-facilitated perovskites.^83^ Luo *et al.* discovered in their research that the exsolution of B-site reducible cations unavoidably resulted in numerous B-site vacancies within the perovskite bulk, which caused unfavorable A-site segregation and a reduction in electronic conductivity.^86^ They proposed a B-site supplement mechanism to mitigate the degradation of perovskite following reduction. Through the precise introduction of foreign Fe ions to supplement the B-site vacancies in reduced Sr_2_Fe_1.3_Ni_0.2_Mo_0.5_O_6−*δ*_, they achieved a robust perovskite scaffold and ensured long-term stable performance.

Compared to directly infiltrating NPs onto the perovskite surface, the embedded structure of *in situ* exsolved NPs within the perovskite matrix confers greater stability. Additionally, *in situ* exsolved NPs exhibit a more uniform distribution on the electrode surface, possess higher particle density, and provide more active sites. Nevertheless, during prolonged operation, these exsolved NPs inevitably coarsen and agglomerate. Consequently, research increasingly focuses on the controllable precipitation of nanoparticles, utilizing methods such as repeated redox manipulations, electrochemical reconstruction, and exsolution energy passivation. These strategies effectively optimize NP size and density, mitigating agglomeration during actual operation. Notably, NP exsolution often coincides with phase changes in the perovskite matrix, which are positively correlated. Further detailed exploration is required to determine whether phase changes in the perovskite influence electrode stability during prolonged operation.

### 3.4 Perovskite-based nanocomposites from self-assembly

In addition to surface modification, the formation of nanocomposites can introduce rich grain boundaries and tailor the lattice strain to induce an effect on the bulk ion conducting properties and surface physicochemical properties, and materials sintering behavior, thus causing an effect on the catalytic performance and durability in the CO_2_RR for the perovskite oxides in SOECs.

Self-assembly is widely used in the creation of functional perovskite-based nanocomposites for different applications. The self-assembly strategy involves initially uniformly mixing metal precursor liquids in each phase, following a calculated stoichiometric ratio, to form a gel-like precursor. Subsequently, the gel-like precursor is subjected to high-temperature calcination, inducing cationic self-assembly into thermodynamically stable multiphase composites. These phases exhibit uniform distribution, close three-dimensional connectivity, abundant TPBs, and facilitate the establishment of rapid ion/electron transmission pathways during electrochemical reactions.^87^ For example, He *et al.* fabricated a Ni@Ni-SFM/Ni-GDC cathode *via* a one-pot synthesis approach.^88^ Based on DFT calculations, they demonstrated superior CO_2_ adsorption capacity and enhanced catalytic conversion ability of Ni@Ni-SFM and Ni@Ni-GDC compared to pristine SFM. Employing Ni@Ni-SFM/Ni-GDC as the cathode, the SOEC attained a remarkable current density of 1.72 A cm^−2^ at 800 °C and 1.5 V. Recently, Shao *et al.* employed a one-pot method for synthesizing Cu_*x*_–Sr_2_Fe_1.5_Mo_0.5_O_6−*δ*_–Gd_0.1_Ce_0.9_O_1.9_ (Cu_*x*_/SFM–GDC) and utilized it as a cathode in SOECs for direct electrolysis of CO_2_.^89^ Experimental evidence has demonstrated that Cu_*x*_/SFM–GDC exhibited outstanding capabilities in CO_2_ adsorption and activation, significantly enhancing the O^2−^/e^−^ conduction rate during the CO_2_RR process. In comparison to the traditional physical hybrid SFM–GDC cathode, the Cu_*x*_/SFM–GDC demonstrated a significantly higher current density of 2.22 A cm^−2^ at 850 °C and 1.6 V, marking a notable increase of 65.7%.

Nanostructured composite materials, prepared through self-assembly, offer a promising alternative to multiphase electrodes produced by physical mixing methods such as ball milling. Self-assembly composites can be efficiently achieved *via* a single heat treatment at relatively low sintering temperatures, resulting in enhanced interfacial areas. Furthermore, the self-assembly technique presents advantages such as heightened catalytic activity, excellent chemical and physical phase compatibility, and robust structural stability.^90,91^ Despite these benefits, the precise design principles governing the self-assembly process remain incompletely elucidated. Further in-depth investigations into reaction mechanisms are imperative to facilitate the rational development of superior heterogeneous electrodes.

### 3.5 Morphology-controlled perovskites

In addition to the compositional tailoring and surface modification, or the formation of nanocomposites, morphology control is also an effective way to optimize the catalytic activity of perovskite catalysts for the CO_2_RR in SOECs, since the microstructural characteristics of the electrode can also greatly impact the performance of SOECs. During operation, the sintering and agglomeration of particles within the electrode would result in loss of TPB and exert adverse effects on the overall cell performance. Fiber materials fabricated *via* electrospinning exhibit a distinctive three-dimensional network structure, which is favorable for efficient gas diffusion within the electrode. Moreover, this distinct structure boasts a higher specific surface area, consequently offering a greater number of active sites for the CO_2_RR and enhancing the catalytic activity of the electrode.^92^ Their unique structure and morphology can also result in a fast exsolution of metal NPs. Furthermore, the distinctive structure, when coupled with exsolution engineering, enables the swift exsolution of metal NPs. For example, Ahmedjonov *et al.* fabricated a La_0.6_Sr_0.4_Co_0.15_Fe_0.8_Pd_0.05_O_3−*δ*_ (H-LSCFP) nanofiber framework electrode *via* electrospinning technology, subsequently crushing and integrating LSCFP into the nanofiber framework, resulting in the formation of hybrid nanofiber electrodes.^93^ By using a Focused Ion Beam-Scanning Electron Microscopy (FIB-SEM) dual-beam system and cutting edge 3D reconstruction techniques to digitally twin two nanofiber electrodes, they established a correlation between the microstructural attributes of the fuel electrode and its CO_2_ electrolysis efficacy. Crushed nanofibers were added to the original nanofiber matrix, which resulted in a significant increase in the volume fraction (48.7%) and tortuosity factor (2.4). This improved O^2−^ transport within the H-LSCFP and improved the CO_2_ surface adsorption/desorption kinetics of the H-LSCFP. Besides, a honeycomb structure with straight channels prepared by freeze casting has also been used in anode-supported SOECs.^10^

In comparison to alternative thin film preparation technologies, atomic layer deposition (ALD) offers distinct advantages in nanomaterial synthesis, such as uniform and conformal deposition onto the substrate, along with meticulous control over the thickness of the deposited layer at the atomic level.^94^ Uncontrolled chemical reactions are prevented due to their self-limiting behavior.^95^ Moreover, the layers deposited *via* ALD exhibit notable consistency and repeatability in surface features. Common metals/oxides have been explored to overcome the issues associated with the catalytic activity of electrodes in fuel cells.^87–90^ The thickness of the catalyst layer is an essential factor as the increased bulk charge transport resistance in the thicker ALD.^96,97^ For example, Pandiyan *et al.* deposited Pt NPs (18 nm) on the surface of La_0.75_Sr_0.25_Cr_0.5_Mn_0.5_O_3_ (LSCrM) perovskite oxides *via* ALD, boosting the electrocatalytic properties.^98^ Nevertheless, the Pt NPs deposited on the LSCrM are prone to agglomeration during annealing, which is not conducive to the long-term stable operation of the cell.

While morphology-controlled methods have demonstrated the ability to increase the contact area with CO_2_, achieving precise control over the preparation process remains a significant challenge. An alternative strategy worth exploring is the development of single-phase oxides with exceptional catalytic activity to advance the industrialization of SOECs for CO_2_ electrolysis.

## 4. Numerical modeling assisted cathode materials development

### 4.1 Machine learning

**Fig. 7:**
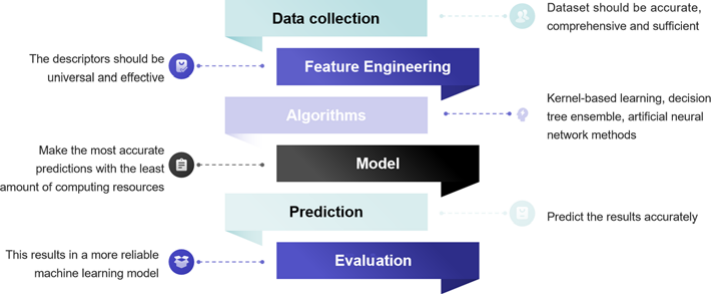
The flow chart of ML in screening catalytic materials.

The conventional exploration process of material design based on experimental studies is extremely costly and time-consuming. Predicting material quality by machine learning (ML) has been shown to be a greatly helpful technique for screening materials, which complements the role of statistics in scientific research.^99^ ML is an efficient and practical approach for predicting and comprehending the catalytic activity, optimal composition, active sites, and CO_2_RR pathways of materials.^100^ As depicted in Fig. 7, the process of ML is like emulating the human learning process, wherein the system scrutinizes a vast dataset through algorithms to construct models and render assessments on novel data.

In ML research, a subset of the data is kept aside to evaluate the reliability of the model, with the remaining data being used to train the model (generally following a 1 : 4 ratio). To create the database, Jacobs *et al.* gathered 749 data points from 313 studies that included 299 distinct perovskite compositions.^101^ Considering that biases arising from various experimental conditions during data collection can directly impact ML outcomes, it is imperative to conduct a comprehensive analysis of the underlying statistics for each catalytic property in the database. This analysis aims to mitigate or eliminate these biases, thereby ensuring the reliability of ML outcomes. In contrast to experimentally acquired data, leveraging DFT calculations to furnish training data for ML models offers advantages such as low data acquisition costs and expedited acquisition of substantial datasets. While the Inorganic Crystal Structure Database (ICSD) and Crystallography Open Database (COD) offer abundant material structure information for ML applications, the primary deficiency in the realm of SOECs lies in the scarcity of extensive and dependable electrochemical datasets on materials. To effectively investigate protonic ceramic fuel cell (PCFC) cathodes with a minimum dataset, Ni *et al.* suggested a unique method based on the experimental design paradigm (EDP),^102^ which facilitated efficient screening and discovery of novel materials by meticulously selecting elements and design matrices, even in the absence of extensive datasets. Furthermore, the *D*_chem_, *k*_chem_, area-specific resistance (ASR), and EC response parameters of materials like BaCo_0.667_Ce_0.167_Fe_0.083_Y_0.083_O_3−*δ*_ and Ba_0.5_Sr_0.5_Co_0.8_Fe_0.2_O_3−*δ*_ predicted by EDP are highly consistent with the experimental results, providing further validation of the model's reliability. Presently, there are two primary types of learning algorithms for supervised learning, in which we instruct the computer on specific tasks, and unsupervised learning, where the computer learns autonomously. In supervised learning, the selection of electrochemical-related features can profoundly impact the model's performance.^99^ Therefore, feature engineering is central to the investigation of catalytic materials. By integrating diverse features, ML models can adeptly capture and predict the properties of perovskite electrodes. Furthermore, converting features into descriptors, which should be universal and effective, is necessary. For perovskite oxide electrodes, the crystal structure, and bulk oxygen p-band center, along with fundamental physical and chemical properties, constitute relevant feature parameters that influence the electrochemical performance of the materials.^103^ For the CO_2_RR, the adsorption energy of CO_2_ on the perovskite surface, the structure features of adsorbates, and the active sites are also important descriptors.^104^ Hence, the selection of suitable descriptors to elucidate structure–activity relationships is paramount for ML in the realm of SOECs. With the progress of deep learning, descriptors are no longer exclusively provided by researchers but can also be automatically acquired through the amalgamation of deep learning with high-performance computing, facilitating the acquisition of suitable descriptors.^100^ More specifically for SOFCs/SOECs, appropriate descriptors such as ASR values and ionic Lewis acid descriptors can further enhance the performance of ML models.^101,105^ Choosing a suitable ML algorithm is an essential task. The ideal model should aim to maximize computational accuracy while minimizing resource consumption. Among the various types of models, kernel-based learning methods, decision tree ensemble methods, and artificial neural networks are widely used mathematical models in ML. For example, Ni *et al.* implemented 8 different regression methods to fit the prepared dataset to predict the ASR values of unexplored materials. Ultimately, four oxides were successfully predicted and confirmed to have superior electro-activity.^105^ One has the option to employ a diverse range of algorithms for data training, followed by the selection of the most optimal algorithm based on predictive accuracy. McGuinness *et al.* used a variety of ML methods including partial least squares (PLS), support vector machine (SVM), principal component regression (PCR), local weighted regression (LWR), multiple linear regression (MLR), artificial neural network (ANN), and gradient-boosted decision tree (GBDT) to predict the thermal expansion coefficients (TEC) of AA′BB′O_3_-type perovskites.^106^ After model training, one crucial step is to evaluate for validation and optimization. Regression problems are commonly assessed using metrics like mean absolute error, root mean squared error, and coefficient of determination. Error rate, accuracy, balanced *F* score, and other metrics are typically used to assess model performance in classification algorithms.^107^ As an efficient and effective ML algorithm, the extreme learning machine algorithm was designed to predict the performance of H_2_O and/or CO_2_ electrolysis of the SOECs.^108^

The design of the cathode material in SOECs plays a crucial role in high-temperature electrocatalytic CO_2_ reduction technology, significantly influencing the efficiency of the CO_2_ reduction reaction. The adoption of ML algorithms for the analysis of extensive datasets facilitates the identification of essential catalytic properties, material optimization, and the exploration of potential reaction mechanisms, thereby advancing the development of perovskite-based cathode materials. The availability and quality of raw data in databases are fundamental for ML training purposes. However, accurately capturing every electrochemical step occurring on the catalyst surface and within particles poses challenges under practical operating conditions. In particular, obtaining *in situ* experimental data at high temperatures is challenging. Therefore, the selection of utilizing DFT calculations to construct a catalyst database for screening the most promising candidate catalysts can be considered as a favorable approach. Based on varying structures, one can calculate properties relevant to catalytic reactions, including band structure, adsorption energy, electronic structure, reaction barriers, and more. By quantifying the relationship between each variable and electrolysis performance, further screening of these descriptors can provide guidance for material design and synthesis. Until now, there has been limited research on the application of ML in SOECs for CO_2_ electrolysis. Hence, enhancing existing databases, descriptors, and their refinement is crucial for enhancing the decision-making capabilities of cognitive systems.

### 4.2 DFT calculations

Combining DFT calculations with experiments has been an increasingly common strategy for perovskite design and discovery. DFT calculations have been extensively employed to corroborate experimental results and elucidate the CO_2_RR mechanism within SOEC cathodes, thereby furnishing a crucial reference for the meticulous design of cathodes. CO_2_ adsorption and dissociation reactions are significant for efficient CO_2_RR. Li *et al.* calculated the relative energy profiles for CO_2_ adsorption and decomposition on the surface of Sr_2_Fe_1.5_Mo_0.5_O_6−*δ*_F_0.1_ (F-SFM), revealing that F-SFM exhibited a lower CO_2_ adsorption and dissociation energy compared to SFM. Based on a series of theoretical calculations, the fundamental steps of the complete electrochemical CO_2_RR cycle within the F-SFM cathode were subsequently proposed.^109^ Kozokaro *et al.* employed DFT and nudged elastic band methodologies to discern the optimal active sites for CO_2_ adsorption on the perovskite surface and demonstrated the significance of oxygen vacancies in the catalytic process.^14^ The energy calculation for CO_2_ adsorption revealed that CO_2_ adheres to the oxygen atoms on the BO_2_ plane *via* the O_surface_–C bond, establishing a robust surface connection through chemical adsorption as CO_3_^2−^. Ye *et al.* employed the DFT method to investigate the CO_2_RR on the surface of SDC surfaces with exsolved Fe clusters (Fe-SDC).^110^ Analysis of the Density of States (DOS) revealed that Fe doping enhanced the d-band center of Ce in SDC, thus augmenting CO_2_ adsorption and activation. Bader charge analysis confirmed that CO_2_ adsorption on Fe clusters transpired more readily than on the SDC surface, accompanied by a lower energy barrier for CO desorption from Fe clusters. Furthermore, it was discovered that the highly coupled gradient of 3d–2p–4f in Fe–O–Ce facilitated the generation of Ce^3+^–Ce^4+^ redox pairs, consequently enhancing the electronic conductivity of Fe-SDC. Ultimately, these enhancements expedited the CO_2_RR on the surface of Fe-SDC. Furthermore, theoretical calculations have revealed that the CO_2_RR was influenced by the metal/electrolyte interface. Consequently, the composition, density, and size of NPs emerge as pivotal factors in shaping the CO_2_RR.^111^ Moreover, J. Payne *et al.* conducted a DFT-based thermodynamic analysis, which corroborated the mechanism governing dissolution, NP nucleation, and perovskite structure evolution. The surface energy scans that were obtained suggest that the Ir^3+^ ions freely moving on the ideal STO surface will be captured by Sr vacancies, since the Ir ions to move away from the 
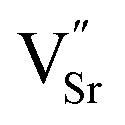
 need to overcome a high energy barrier (≈5 eV). Subsequently, these captured ions can pair with other mobile Ir ions forming a stable Ir–O–Ir under the mediation of surrounding O, potentially serving as initial nucleation sites for Ir cluster growth and reduction.^112^

Sr surface segregation in perovskites may lead to degradation of the electrode performance. Numerous studies on SOFCs have demonstrated that Sr-containing perovskites undergo segregation during high-temperature operation, resulting in surface enrichment and the formation of various product phases, including SrO and SrCrO_4_.^113^ During SOEC operation, these segregated A-site metal oxides have demonstrated a significant enhancement in the CO_2_ adsorption for the CO_2_RR on perovskite oxide cathodes, but an optimal doping amount still exists, and the stronger adsorption of carbonate could lead to complete reduction of CO_2_ to form carbon during electrolysis.^114^ Kozokaro *et al.* calculated the energy of Sr segregation with three different amounts of oxygen surface vacancies on the LSFCr surface. The calculations showed that the reduction of CO_2_ to CO and the formation of SrCO_3_ were competitive reactions on the same LSFCr, and therefore O vacancies were needed not only to improve catalysis but also to block the competing reaction of SrCO_3_ adsorption.^14^

## 5. Advanced characterizations for the fundamental understanding of CO_2_ electrolysis in SOECs

The in-depth understanding of the fundamentals in CO_2_ electrolytes over the SOEC anode is of critical importance to provide useful guidance for the design and optimization of perovskites and other materials to realize highly efficient, durable CO production from electrochemical reduction of CO_2_ at elevated temperature. In the past few years, various advanced *in situ* characterization techniques have been developed, as listed in Table 2, which are extremely helpful for understanding cell degradation mechanisms, probing the underlying mechanisms of CO_2_RR, and ultimately providing guidance in designing new advanced materials for accelerating commercialization. Characterizing the electrode state under operating conditions, particularly for high-temperature SOCs, presents significant challenges. Such characterization necessitates the consideration of numerous factors, including temperature, applied voltage, and the atmospheric conditions of the two electrodes, among others.

**Table 2:** Summary of advanced characterization techniques applied to the cathode in SOCs

Materials	Techniques	Issues/challenges	Ref.
La_0.66_Ti_1−*x*_Fe_*x*_O_3−*δ*_ (*x* = 0, 0.1, 0.2, 0.3, 0.4)	• *In situ* XRD & NAP-XPS & ^57^Fe Mossbauer spectra	• Phase stability under a simulated electroreduction atmosphere	115
Cu/YSZ	• NAP-XPS & NEXAFS	• The evolution of the status of Cu thin film electrodes in contact with YSZ during the CO_2_ reduction reaction	116
(La,Sr)FeO_3−*δ*_, (La,Sr)CrO_3−*δ*_	• NAP-XPS	• Revealed the formation of a carbonate intermediate	117
Fe_0.05_Sm_0.2_Ce_0.8_O_2−*δ*_	• UV Raman spectra & XAS	• Confirm the oxygen vacancies and the change in the valence of Fe	118
Ru1/SDC-La_0.6_Sr_0.4_Co_0.2_Fe_0.8_O_3−*δ*_	• *In situ* XPS & XAS & PALS	• Demonstrate the presence of single Ru atoms on the SDC surface	62
Sr_2_Fe_1.4_5Ir_0.05_Mo_0.5_O_6−*δ*_	• *In situ* NAP-XPS & *in situ* XAS & *in situ* XRD	• Exsolution mechanism of Ir and Fe on the SFIrM surface using the *in situ* electrochemical method	80
Nanofiber La_0.6_Sr_0.4_Co_0.15_Fe_0.8_Pd_0.05_O_3−*δ*_	• 3D reconstruction technique with a FIB-SEM dual beam system	• Illustrates the microstructure of the electrode	93
La_0.7_Sr_0.2_Ni_0.2_Fe_0.8_O_3−*δ*_	• *In situ* XRD & *in situ* TEM & *in situ* TPD-DRIFTS & XAS	• Structural transformations under a reducing atmosphere	119
La_0.6_Sr_0.4_Co_0.7_Mn_0.3_O_3−*δ*_	• *In situ* XRD & XAS	• Determine the average oxidation state of the B-site element	120
Pr_0.1_Ce_0.9_O_2−*δ*_	• AP-XPS & AP-XAS	• Quantify the differences between surface defect chemistry of PCO and the bulk counterpart	121

### 5.1 X-ray techniques

**Fig. 8:**
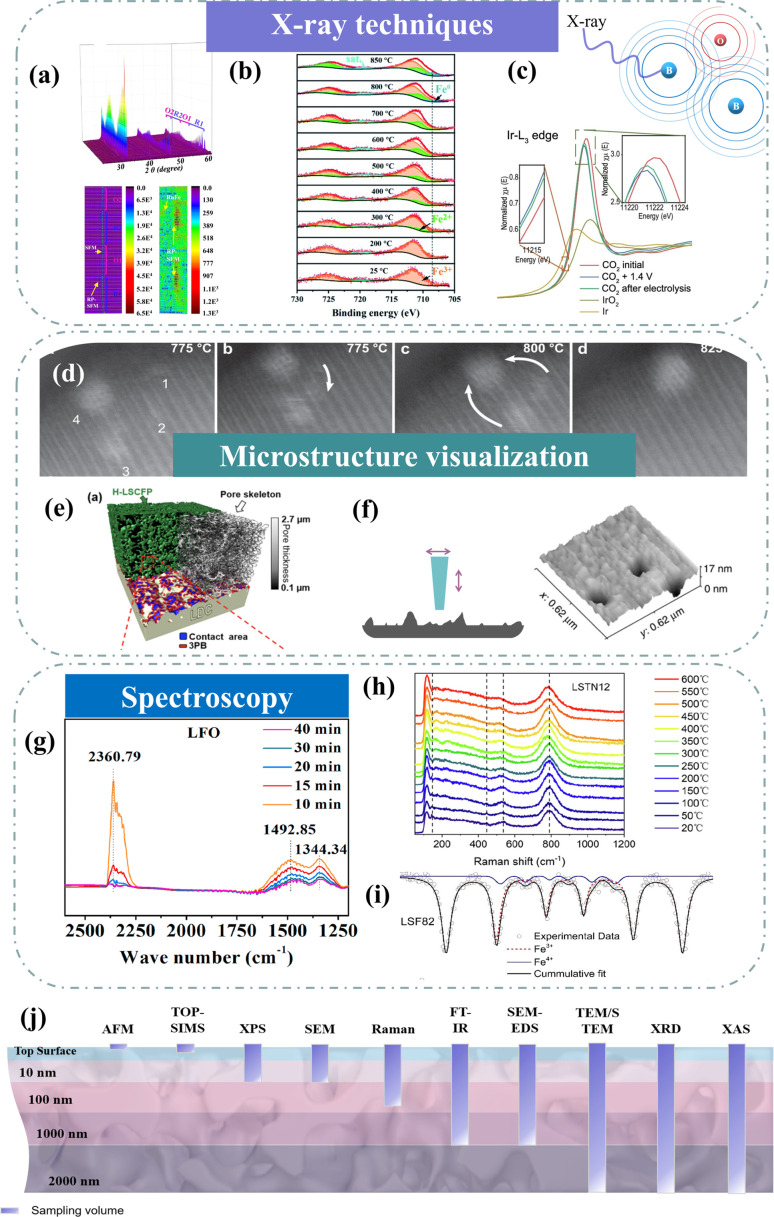
(a) *In situ* XRD patterns of SFRuM upon switching between a reducing and oxidizing atmosphere at 800 °C and corresponding depth profile of XRD patterns. Reproduced from ref. 78. Copyright 2021, Springer Nature. (b) *In situ* XPS. Reproduced from ref. 115. Copyright 2020, The Royal Society of Chemistry. (c) *In situ* Ir L3-edge XANES spectra. Reproduced from ref. 80. Copyright 2023, Oxford University Press. (d) STEM micrographs of grains monitored *in situ*. Reproduced from ref. 112. Copyright 2023, Springer Nature Limited. (e) 3D reconstructed architecture image. Reproduced from ref. 93. Copyright 2024, Springer Nature. (f) AFM image. Reproduced from ref. 73. Copyright 2015, Springer Nature Limited. (g) Quasi *in situ* IR absorbance spectra. Reproduced from ref. 122. Copyright 2021, Elsevier. (h) *In situ* high-temperature Raman spectra. Reproduced from ref. 123. Copyright 2023, Elsevier. (i) Mössbauer spectra. Reproduced from ref. 114. Copyright 2021, Elsevier. (j) Schematic of typical analysis depths for techniques.

X-ray diffraction (XRD) has demonstrated its importance in characterizing physical and chemical properties. Targeting the high temperature of SOC technologies, high-temperature *in situ* XRD is one of the most attractive methods to reveal structural and chemical information.^107–109^ Through high-temperature *in situ* XRD, we can see how the crystal structure of the electrode changes in the temperature range from room temperature to working temperature. For example, Wang *et al.* utilized *in situ* XRD technology to visually capture the exsolution and subsequent disappearance of the RuFe alloy within Sr_2_Fe_1.4_Ru_0.1_Mo_0.5_O_6−*δ*_ (SFRuM), achieved through cyclic switching of the redox atmosphere at 800 °C.^78^ Concurrently, detailed analysis of the peak intensity variations in *in situ* XRD indicated that repeated redox operations significantly facilitated the exsolution of the RuFe alloy (Fig. 8a). The dynamic evolution of the structure of Sr_2_Fe_1.45_Rh_0.05_Mo_0.5_O_6−*δ*_ (SFRhM) during redox activity has also been investigated with *in situ* high-temperature XRD.^124^ After high-temperature reduction, the double perovskite (SFRhM) gradually evolved into layered perovskite (RP-SFRhM), accompanied by the emergence of RhFe alloy. During the reoxidation process, the RhFe alloy phase gradually disappeared, and the RP-SFRhM recovered to pure SFRhM. The voltage-driven exsolution method for *in situ* electrochemical reconstruction is an efficient way to produce abundant nanostructures faster than the conventional reduction by hydrogen.^84^ Investigating the dynamic structure evolution to understand the correlation between structure and the CO_2_ electrolysis process is imperative. By applying a voltage that increases in steps, the Ruddlesden–Popper perovskite (RP-SFIrM) and the IrFe alloy phase could be observed respectively.^80^

X-ray photoelectron spectroscopy (XPS) is an effective surface analysis technology to monitor surface metal valence states and surface oxygen vacancies by fitting peaks.^83^ However, because of the intense interaction between the photoelectrons ejected and gas phase molecules, conventional XPS needs to be carried out in high vacuum or ultra-high vacuum settings.^125^ These are far from the real working conditions. *In situ* near ambient pressure XPS (NAP-XPS) measurements were used to investigate the exsolution of NPs and adsorbed carbonate species, revealing numerous details of the CO_2_ electrolysis. Under the simulated electroreduction environment, it was observed by NAP-XPS (Fig. 8b) that Fe^3+^ in La_0.66_Ti_0.8_Fe_0.2_O_3−*δ*_ (LTF2) began to reduce to Fe^2+^ at 300 °C, and Fe^0^ appeared at 800 °C.^115^ Under cathodic polarization, inferring possible CO_2_RR mechanisms based on the evolution of carbon species represents a prominent subject of discussion. Opitz *et al*. employed NAP-XPS to visually unveil the formation of carbonate intermediates during cathodic polarization, subsequently engaging in a comprehensive discussion on the CO_2_ electrolysis mechanism at the perovskite surface.^117^

X-ray absorption spectroscopy (XAS), which reflects the electronic transition from the inner shell to the valence band, provides valuable information on the oxidation state, site symmetry, bond strength, coordination number, bond distances, and identities of nearest neighbor atoms. X-ray Absorption Fine Structure (XAFS) spectra are commonly separated into the X-ray Absorption Near Edge Structure (XANES) and the Extended X-ray Absorption Fine Structure (EXAFS).^126^ Typically, the oxidation state of an element and its alterations are primarily ascertained through the displacement of the main absorption edge. XANES spectroscopy discerns the oxidation state of pertinent elements in unfamiliar materials through spectral comparison with similar oxidation states of the same atoms. Kim *et al.* indicated the increase of Fe–Fe shell intensity from the Fourier-transformed EXAFS spectra, confirming the exsolution of Fe NPs onto the surface after reduction.^127^ The development of the single atom synthesis strategy has matured significantly. On the other hand, the primary difficulty with SACs is the aggregation of metal atoms at high temperatures (600–800 °C). It is a challenge to verify the formation of single atom species using atomic-resolution characterization techniques. Instead of the Pt–O correlation observed in model compounds (such as Pt metal, Pt acetylacetonate, and PtO_2_), it was conjectured that the predominant peak in the Fourier transform of Pt L3-edge EXAFS spectra of Pt/ceria stemmed from Pt–O–Ce. Additionally, the presence of Pt single atoms was validated through fitting and comparative analysis of the EXAFS data from Pt/ceria with the theoretical spectra of Ce_1−*x*_Pt_*x*_O_2−*δ*_ type solid solution and Pt metal. Based on the distinction of the coordination number of 8 for a cubic fluorite structure, the presence of Pt single atoms was confirmed.^61^ Specifically, XAS is suitable at low concentrations (0.005–10 wt%) and a broad range of testing conditions (−269–1200 °C) from vacuum to high pressure. *In situ* XAS measurements provide strong evidence of the chemical state and coordination environment. Shen *et al.* observed that during the CO_2_ electrolysis process at 1.4 V, there was a reduction in the intensity of the white line peak at the Ir L3-edge. The formation of an IrFe alloy was demonstrated in both cases.^80^ X-ray diffraction (XAS) has emerged as a potent method for obtaining the electronic structure, local coordination environment, and oxidation states, which are critical to investigating electrocatalysts.^128^ However, the uncertainty in fit parameters can be 10–15%, and the insensitivity of minor species in mixtures is also noteworthy. Hence, XAS results and interpretations do not stand alone. Without additional information, analysis of the contributions of different species (different oxidation states, particles of different sizes and composition) in these cases is extremely challenging.^129^

### 5.2 Microstructure visualization of electrodes

*In situ* characterization technology enables real-time observation of the exsolution and growth mechanism of NPs on the electrode, the dynamic evolution of the solid–solid contact interface between the electrode and the solid electrolyte under prolonged high-temperature conditions, and the microstructural state of the material during device operation.^112,127,130–132^

*In situ* transmission electron microscopy (*in situ* TEM) is widely recognized as a powerful characterization technique that enables real-time observation of electrochemical reactions with high spatial resolution (atomic scale) and temporal resolution (subsecond). *In situ* exsolution is a highly effective strategy for CO_2_ electrolysis in SOECs. Comprehending the exsolution process not only enables better adjustment of activity and stability but also offers valuable guidance for developing abundant interfaces with enhanced functionality. Neagu *et al.* employed *in situ* TEM to capture pictures at various points in time of the progressive development of a particle socket and nucleation of two more particles from a powder sample of La_0.43_Ca_0.37_Ni_0.06_Ti_0.94_O_3_ at 900 °C in an H_2_ (20 mbar) environment. Rather than the few hours reduction durations commonly reported in the literatures, the majority of the particle growth occurred in less than 100 s.^133^ Neagu *et al.* further elucidated that the diffusion of Ir originated from SrIr_0.005_Ti_0.995_O_3_ bulk and the amalgamation of mobile metal clusters proceeded from the atomic scale to their ultimate nanoscale configuration through *in situ* high-resolution STEM observations (Fig. 8d).^112^

The correlation between structure and performance is crucial for optimizing the design and efficiency of CO_2_ electrolysis systems. The 3D reconstructions obtained by FIB-SEM tomography can offer valuable insights into the relationship between the microstructural features of the fuel electrode and its CO_2_ reactivity.^134^ Analyzing these reconstructions will help in understanding how the electrode's physical characteristics influence its functionality in the electrolysis process. Following a duration operation at 800 °C in a CO/CO_2_ atmosphere with varying constant current densities (0 and −1 A cm^−2^), FIB-SEM tomography was used to reconstruct the microstructure development of Ni/YSZ-supported planar-type SOECs.^135^ Akhmadjonov *et al.* demonstrated the phenomenon of nickel migration at the electrolyte/electrode contact during long-term CO_2_ electrolysis, suggesting that the current was a significant factor in this process. The durability of the hybrid structured nanofiber-based fuel electrode was further confirmed by quantified 3D microstructural characteristics based on parameters like volume fraction, tortuosity coefficient, connectivity, surface area, and TPB value analyzed in the total volume of the electrode sample (Fig. 8e).^93^

An atomic force microscope (AFM) is a powerful tool used to visualize and manipulate materials at the nanoscale level. Similar to lightly gliding your fingertip along the surface of an item, it measures the forces between the tip and the atoms on the sample surface by scanning a sharp tip across it.^136,137^ Surface pitting occurred when concentrated HNO_3_ was used to etch La_0.52_Sr_0.28_Ni_0.06_Ti_0.94_O_3_. The number density and size distribution of these pits were comparable to the exsolved particles, suggesting that the exsolved particles were deeply entrenched in the perovskite bulk. This finding was demonstrated in research by Neagu *et al.* using AFM (Fig. 8f).^73^ The large coverage of the Sr-rich phase of the surface with the segregated particles can also be detected by AFM.^132,138^

### 5.3 Spectroscopy: Raman, IR and Mössbauer

Taking advantage of the fingerprinting of molecules based on their vibrational modes, infrared spectroscopy (IR) is a powerful technique for identifying adsorbed species on electrode surfaces. The majority of studies based on IR on the CO_2_RR have identified CO_3_^2−^ as a common intermediate. The infrared band ranging from 2380 to 2300 cm^−1^ is attributed to the adsorption of CO_2_ molecules on the perovskite surface, whereas the band spanning from 1450 to 1380 cm^−1^ is associated with CO_3_^2−^.^6,139^*In situ* IR absorbance spectra were recorded during CO_2_ desorption to comprehend the CO_2_ adsorption configuration on cathode surfaces.^131–133^ During desorption, the peak intensity of the 2360.79 cm^−1^ absorption peak associated with the C=O vibration of the CO_2_ molecule and the 1600–1300 cm^−1^ absorption vibration frequency double peak related to the tridentate carbonate species gradually decreases (Fig. 8g).^122^ In addition, *in situ* infrared can also be used to test the stability of the adsorbed CO_2_ on the Sr_2_FeMo_2/3_Mg_1/3_O_6_ (SFMM-R) sample at elevated temperatures between 200 and 700 °C.^140^

Raman spectroscopy is a unique chemical fingerprint of specific molecules or materials, which can be used to quickly confirm the type of material or distinguish between different materials.^141^ Typically, Raman has been successfully used to detect whether there is carbon deposition on the cathode after stability testing due to the reasonable sensitivity of Raman spectroscopy to carbon species.^142^ Materials with centrosymmetric crystal structures like cubic metals (BCC or FCC) and perfect perovskites (ABO_3_) cannot generate Raman scattering.^143^ This property makes Raman spectroscopy a valuable tool for analyzing changes in lattice symmetry due to factors such as doping, element segregation, contamination, and redox reactions in SOEC electrodes. *In situ* exsolution of Ni NP-supported (La_4_Sr_*n*−4_)_0.9_Ti_0.9n_Ni_0.1*n*_O_3*n*+2_ (*n* = 5, 8, 12 (LSTN*n*)) is a potential CO_2_ electrolysis catalyst. Under reducing conditions of 5% H_2_/95% N_2_ from 20 °C to 600 °C, *in situ* Raman spectroscopy revealed a gradual weakening in the rotational vibration of the TiO_6_ octahedron of LSTN*n* along the *c*-axis (Fig. 8h).^123^ This is because oxygen atoms depart from the lattice of the BO_6_ octahedron and nickel segregates from the lattice, leading to a significant contraction in lattice parameters.

Mössbauer spectroscopy has been widely used as a tool for fundamental research in solid-state physics, especially to differentiate between various species of iron.^139,144^ Iron-based perovskites have been universally used in the development of cathodes for SOECs, and iron is also often involved in the electrolytic CO_2_ reaction as an active site. The Mössbauer method was often used to monitor the oxidation state of Fe following various long-term operations.^145^ Mahmoud *et al.* compared the initial state of the cell with the Mössbauer spectra data acquired for 0, 1774, 6100, and 9000 h of operation to better understand the structural and electrochemical changes that occur after an operation.^146^ After prolonged operation, the Mössbauer spectrum results revealed the reduction of iron from perhaps pentahedral Fe(iv) to octahedral Fe(iii). After 9000 hours of operation, Co_3_O_4_ NPs appeared in the La_0.58_Sr_0.4_Fe_0.5_Co_0.5_O_3−*δ*_ electrode, and the Fe(iv) concentration decreased from 18% to 11%. According to the different A-site occupancies, the carbon formation of LSF (La_0.8_Sr_0.2_FeO_3−*δ*_) will also be different in the electrolysis process of CO_2_ (Fig. 8i).^114^ Mössbauer spectra of La_0.7_Sr_0.2_FeO_3−*δ*_ (LSF72) and La_0.9_Sr_0.2_FeO_3−*δ*_ (LSF92) collected at room temperature show that the LSF72 crystal structure exhibited a broader range of iron species types compared to LFS92. The oxidation state of Fe in the perovskite decreased with an increase in A-site occupancy.

Advanced *operando* techniques are essential to meet the scientific challenge (Fig. 8k). The new insights they offer have contributed to a mechanism understanding of degradation and real-time monitoring of carbon deposition on the electrode and exsolution of NPs, and are guiding future directions for material optimization and innovation.

## 6. Pre-industrial application trials

The electrolysis of CO_2_ using SOEC technology is a promising approach for efficiently converting CO_2_ into valuable fuels and chemicals. This process contributes to carbon capture and utilization, offering a potential avenue to mitigate greenhouse gas emissions. The choice of cathode is pivotal in determining the overall performance, efficiency, stability, and economic viability of SOEC systems. When it comes to lower temperature conversions, an SOEC is more efficient at using industrial waste heat than other electrochemical techniques. Since part of the energy is derived directly from heat, this eliminates the intermediate process of converting heat to electricity and improves energy utilization. While there are many potential uses for high-temperature electrolysis technology in the chemical sector, stack performance and degradation may differ significantly from single cell studies. There are still many key technological challenges that urgently require breakthroughs to achieve large-scale commercialization.

**Fig. 9:**
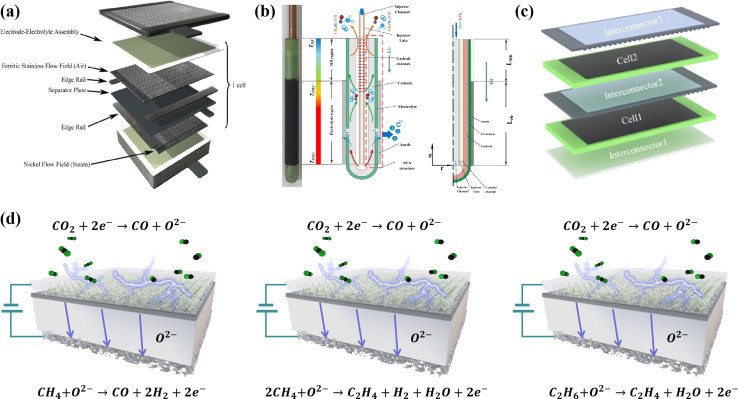
Various SOEC stack structures: (a) planar. Reproduced from ref. 148. 2013, Elsevier. (b) Tubular. Reproduced from ref. 149. 2021, Elsevier. (c) Flat tubular. Reproduced from ref. 150. 2023, Elsevier. (d) Schematic of the working principle of an electrochemical cell fueled with different raw materials.

The main structures of SOECs adopted at this stage are planar structure, tubular structure, and flat tubular structure^140,141,147^ (Fig. 9a–c). The structure of a planar SOEC stack includes electrolysis cells, many accessories such as a current collector plate, interconnect, seal layers, and necessary components such as a heat management system, purification system, and automatic control system.^151^ Since it is easy to achieve the airtight sealing and interconnection of single cells in the manufacturing process of tubular SOECs, compared to planar SOECs, tubular SOECs have certain advantages, but they also face issues such as complex manufacturing processes, low power density, and high manufacturing cost. More recently, micro-tubular and cone-shaped structures have been studied and developed. However, the problems with the planar structure and the tubular structure do not seem to have been completely solved for these designs.^152^ To address the issue of substrate edge warping in planar structures during long-term operation, researchers have developed a flat tubular structured SOEC by integrating the geometric symmetry characteristics of tubular structures. The flat tubular structure, which amalgamates the benefits of both flat and tubular structures, demonstrates commendable performance and mechanical properties. Furthermore, it is more convenient to configure as a stack compared to a traditional tubular structure. Nevertheless, challenges persist, including the high costs associated with stacks and systems, as well as the limited robustness in managing dynamic operations.^153,154^ With the development of novel electrodes, the performance of SOECs has been greatly enhanced. The CO_2_RR on the cathode is largely influenced by the number of reaction sites, the size and distribution of porosity for effective gas transport in the high-temperature reduction environment, and the catalytic activity of the cathode surface. Haldor Topsoe's commercial CO_2_ electrolysis system based on SOEC technology combines CO_2_ electrolysis and gas purification in one unit and is capable of producing 96 N m^3^ high-purity carbon monoxide gas per hour.^155^ Owing to the good electronic conductivity and catalytic activity of Ni metal, cermet composites like Ni-YSZ still stand out as the predominant electrocatalysts utilized in fuel electrodes, having successfully transitioned into widespread commercial usage. Regrettably, the Ni surface operating within carbon-containing fuels is susceptible to carbon deposition and agglomeration, thereby inducing performance degradation over prolonged operational durations.^156^ Zhan *et al.* used a planar Ni-YSZ electrode-supported SOFC to achieve 1 A cm^−2^ at 800 °C and 1.3 V with a combination of 25% H_2_ and 75% CO_2_ inlet gas.^157^ As Ni-YSZ substitutes, numerous perovskites and fluorite oxides were researched. Kaur *et al.* investigated the performance of ((La_0.6_Sr_0.4_)_0.95_Co_0.2_Fe_0.8_O_3−*δ*_)–Gd_0.2_Ce_0.8_O_1.95_ with A-site defects for CO_2_ electrolysis in symmetric tubular cells.^158^ With no extra protective gas used, current densities of LSCF–DC|GDC‖YSZ‖GDC|LSCF–GDC approached 200 mA cm^−2^ at 1.2 V in CO_2_ at 800 °C for 350 h.

The main challenges faced by most perovskite oxide electrodes are the lower electronic ionic conductivity and poor electrochemical catalytic activity. Therefore, the current research focus is on improving the electrocatalytic activity and electronic ionic conductivity of perovskite oxides through methods such as infiltration, doping, and *in situ* exsolution. Employing SOEC high-temperature CO_2_/H_2_O co-electrolysis facilitates the storage of renewable energy in the form of chemical energy, such as syngas or hydrocarbon fuels, thereby boasting potential application scenarios in the realm of energy storage.^159^ Reytier *et al.* performed stack-level studies (10-cell stack) in both electrolysis and co-electrolysis modes.^160^ The configuration of the stack was NiO-8YSZ|8YSZ|CGO|LSC with an active area of 100 cm^2^. The maximum current density applied per cell at 1.15 V voltage is −0.8 A cm^−2^ with an inlet gas composition of 65 vol% H_2_O + 25 vol% CO_2_ + 10 vol% H_2_, the H_2_ + CO syngas production of the stack was 0.34 N m^3^ h^−1^ at −80 A and 800 °C. The concurrent occurrence of H_2_O/CO_2_ co-electrolysis and methanation processes at the cathode further enhanced the advantage of SOEC co-electrolysis. When the temperature decreased to around 600 °C, methane began to form in the electrolysis products. Methane synthesis properties in tubular SOECs (115 mm in length, 5 mm in inner diameter and 6.59 mm in outer diameter) between 550 and 650 °C were investigated by Luo *et al.*^161^ At 550 °C, when a voltage of 1.5 V was applied, the CH_4_ yield reached 12.34%, with an inlet gas composition of 20% H_2_O, 20% CO_2_, 20% H_2_, and 40% Ar.

Synthesis gas itself is one of the major chemicals for various chemical and fuel syntheses in modern industry. Gaseous or more complex chemicals and fuels require further downstream processes after the electrolysis cell. The integration of SOECs with several chemical industrial processes such as the synthesis of ethylene, oxidative dehydrogenation of ethane (ODE), and dry reforming of liquid alcohols have already received attention from researchers and have been studied to some extent.^162–164^

As shown in Fig. 9d, SOECs offer a promising avenue for bridging the energy storage and chemical synthesis industries through electrolysis. Recent studies have demonstrated the feasibility of enhancing the kinetics of the CO_2_ electrolysis reaction and reducing power consumption significantly. Guo *et al.* introduced methane on the SOEC anode side to produce syngas, leading to accelerated reaction kinetics.^165^ Chen *et al.* utilized Sr_2_Ti_0.8_Co_0.6_Fe_0.6_O_6−*δ*_ as a dual anode and cathode material to effectively catalyze the conversion of ethane to ethylene on the anode and the reduction of CO_2_ to CO on the cathode. It provides an important regulatory knob for achieving high catalytic activity of small molecule conversion.^166^ In addition to performance improvement, an essential prerequisite for stacks in large systems is the ability of the stack to perform reliably in real-life situations. Long-term operation of SOECs is susceptible to performance deterioration because of their high operating temperature. In terms of the cathode, poisoning of sulfur, cadmium, and SiO_2_, microstructure degradation, and carbon deposition are the main degradation reasons.^167–171^ Poisoning is caused either by impurities in the steam or the volatile species evaporated from glass-ceramic sealants, while interconnects reduce reaction sites and hinder charge transfer and oxygen ion transport pathways, leading to electrode degradation and electrolysis performance decline. The purity of the inlet gas is very important for extending the life of the SOECs. Furthermore, a major challenge to CO_2_ electrolysis in SOCs is the conflict between carbon deposition (CO_2_ (g) → C + O_2_ (g)) and CO production (2CO_2_ (g) → 2CO (g) + O_2_ (g)). Königshofer *et al.* investigated various kinds and intensities of impacts on the stack's performance at various reactant conversion rates, operating temperatures, and gas mixes. Mitigation measures of steam regeneration have been proposed for degradation caused by carbon deposition.^172^ To illuminate the carbon deposition mechanism, Skafte *et al.* thoroughly studied the relationship between carbon deposition and oxidized carbon intermediates and carbonate coverage.^173^ Furthermore, the Nernst voltage generated on the SOEC was proportional to the driving voltage provided to the SOEC, making voltage play a crucial role in regulating Ni oxidation and carbon deposition. The electrolysis voltage can simultaneously suppress Ni oxidation and carbon deposition in the range of −0.9 V to −1.4 V.^171^ However, the performance degradation caused by nickel coarsening in nickel-based materials remains unavoidable. Potential issues within the Ni cermet fuel electrode encompass Ni particle agglomeration/coarsening and Ni migration, culminating in a notable increase of the series resistance of the cells. The 3D electrode microstructures, after enduring a 1000 hours durability test under CO_2_ electrolysis, were utilized to investigate the mechanism of Ni migration using FIB-SEM tomography. During long-term CO_2_ electrolysis, not only will there be coarsening of Ni particles, but Ni will also migrate away from the electrode/electrolyte interface, resulting in irreversible loss of electrochemical performance.^135^ As a result of stack improvements, degradation rates have dropped to below 0.4% per 1000 h. Meanwhile, the appearance of CO_2_ electrolysis mode operating for over 1 year demonstrates that SOECs are one of the most suitable CO_2_RR technologies for commercialization.^174^

## 7. Summary and outlook

When it comes to storing renewable energy in the form of useful chemicals and fuels, SOECs are seen as one of the best solutions due to their high efficiency with nearly 100% faradaic efficiency and favorable reaction kinetics resulting from elevated operation temperatures. However, the rapid performance degradation, mainly due to the poor durability of state-of-the-art Ni-YSZ based cathodes at high-temperature operating conditions greatly hinders its widespread application. Alternative cathode materials are urgently needed, and the advanced ones should possess certain features to optimize the multiple physical and chemical processes during the CO_2_ electrolysis process, such as a large number of active sites, adequate electronic conductivity, and chemical, thermal, and mechanical stability. Compared to the traditional Ni-YSZ cathodes, perovskite oxide cathodes have demonstrated significant potential when used for direct CO_2_ electrolysis in SOECs. In particular, metallic NPs loaded on perovskite oxide surfaces, through infiltration, assembly, or co-deposition methods, have exhibited excellent catalytic capabilities, but their long-term stability is limited due to weak metal–support interactions.

In this review article, we have provided a thorough review of the current advancements, challenges, and prospects of perovskite-type cathodes for SOECs in CO_2_ electrolysis. It covers a comprehensive summary of recent design concepts for SOECs, aiming to help scholars with more insights into the design of novel cathodes with improved catalytic activity and stability for CO_2_ electrolysis. Emphasis was placed on *in situ* characterization and DFT calculations for an insightful understanding of the mechanism of electrolysis of CO_2_ and analysis of the origins of electrode performance degradation. Additionally, the rise of ML has contributed greatly to the rapid screening of superior candidate materials. However, to achieve a high degree of commercialization on an industrial scale, it is essential to develop new cathode materials that significantly improve durability and stability. Based on the latest research results, here we propose some suggestions for future development directions in perovskite-type cathodes for SOECs towards CO_2_ electrolysis.

(1) Exsolution has emerged as a promising method to fabricate NP-decorated oxides, as the NPs are anchored onto the host oxide with strong mutual interactions. This can suppress particle sintering, ultimately improving the stability of the catalysts. Various strategies involving structural modifications (defects and phases) and control of operating conditions (gas composition, temperature, potential, and strain) are utilized to tailor the size, composition, and distribution of the exsolved nanostructures. Nevertheless, the mechanisms of exsolution are still not fully understood. Actually, there even exist some conflicts in the explanations, like the relationships between the perovskite surface and the exsolved NPs. With the help of advanced characterization techniques and theoretical calculations, we may get a clearer picture of the electronic and atomic structures of the materials, thus guiding the development of new methods for exsolution to precisely control the density and distribution of exsolved NPs.

(2) Currently, single atoms, high-entropy, and other engineering approaches are being widely explored and exhibit great potential in improving catalytic performance. High-entropy perovskite oxides are outstandingly promising materials for the CO_2_RR *via* SOECs, which exhibit favorable performance stability due to entropy stabilization. Especially at high temperatures, such stabilization effect is more obvious considering that the effect of increasing entropy is magnified at higher temperatures based on Δ*G* = Δ*H* − T*Δ*S*. On the other hand, the chemical element variation at the A/B sites of perovskite oxides can also help to tailor the catalytic activity of the materials. This leads to a new consideration about the origin of the improved performance, the high-entropy effect or the multi-element doping effect. Direct evidence about the real contribution is unfortunately still lacking. The unique maximum atom utilization and remarkable high catalytic performance of SACs will undoubtedly attract the attention of researchers. However, the thermal stability of SACs is crucial for their practical application under reaction conditions (including CO_2_ or reducing atmospheres). There is an urgent need to further improve the stability of SACs. Currently, metal loading in SACs is still at low levels; to improve the metal loading and prepare dual atom catalysts, it is necessary to delve deeper into the structure–performance relationship and catalytic mechanisms. This will enable the rational design of superior SACs for high-temperature CO_2_ electrolysis.

(3) Fundamental understanding is the basis of material development. An in-depth understanding of the growth and working mechanisms of NPs based on exsolution from a perovskite lattice is still not available. Credible chemical information on the catalysts is provided by advanced *in situ* characterization technologies, such as XRD, XPS, and XAS. *In situ* Raman and FT-IR are widely used to investigate the mechanism of the CO_2_RR by identifying the carbonate species. Secondary ion mass spectrometry and isotope exchange investigations will develop into potential characterization tools that provide rich information on structural design. Through the combination of theoretical calculations and advanced *in situ* characterization, the pathways of CO_2_ reduction will be provided to understand the correlation of structure and activity during the CO_2_RR. Most researchers may lack the opportunity for *in situ* characterization due to the expensiveness and access limitations of synchrotrons, so when *ex situ* characterization is employed, it should closely approximate the working conditions of the material and provide a thorough explanation of the testing conditions.

(4) ML has great potential in rapid material screening. Predicting catalytic activity, active site identification, and reaction pathways in the CO_2_RR through ML techniques will accelerate the development of high-performance cathodes. In addition, by combining ML with insights obtained from the mechanisms of decayed cell performance, we can understand the primary failure modes and extend that lead to performance degradation. Therefore, establishing a database with a large amount of reliable data and selecting appropriate descriptors are essential to fully leverage the potential of ML.

(5) Scaling SOEC devices to the industrial level is extremely challenging. There has been a large amount of experimental research on single cells. While the current density of single cells (small-area button-type SOECs) can reach 3 A cm^−2^ (800 °C, 1.5 V) or even higher, the output performance of the SOEC stack experiences a substantial decline with the continuous amplification of single cells. The commercialization challenges of SOEC technology lie in its short lifespan, susceptibility to damage, and inability to operate stably for long periods. Particularly at high current densities, the degradation of the cathode is the main reason. Therefore, while optimizing the design of the electrolysis stack system, it is crucial to develop advanced cathodes. Perovskites could be feasible electrodes to replace traditional Ni-YSZ in the future. At the same time, more theoretical simulations and experimental research should be conducted to further confirm the cost-effectiveness and technical viability of coupling high-temperature electrolysis, renewable energy generation, and chemical synthesis processes.

Overall, while the industrialization of SOECs for CO_2_ electrolysis still faces significant challenges, we firmly believe that combining various *in situ* characterizations with advanced theoretical calculations can further clarify the reaction mechanisms of high-temperature CO_2_ electrolysis in complex reaction environments. This will lay the foundation for researchers to design excellent cathodes, eventually making SOECs a practically applicable technology to help humanity realize a net-zero carbon society.

## Data availability

Data availability is not applicable to this article as no new data were created or analyzed in this study.

## Author contributions

Ruijia Xu: writing – original draft preparation & visualization. Shuai Liu: writing – original draft preparation, review & editing. Meiting Yang, Zhixin Luo: visualization & review. Guangming Yang: project administration, supervision, review & editing, and funding acquisition. Ran Ran & Wei Zhou: supervision & review. Zongping Shao: main supervision, review & editing.

## Conflicts of interest

There are no conflicts to declare.
